# Amino acid δ^13^C and δ^15^N analyses reveal distinct species‐specific patterns of trophic plasticity in a marine symbiosis

**DOI:** 10.1002/lno.11742

**Published:** 2021-04-07

**Authors:** Christopher B. Wall, Natalie J. Wallsgrove, Ruth D. Gates, Brian N. Popp

**Affiliations:** ^1^ Hawai'i Institute of Marine Biology University of Hawai'i at Mānoa Honolulu Hawaii USA; ^2^ Pacific Biosciences Research Center University of Hawai'i at Mānoa Honolulu Hawaii USA; ^3^ Department of Earth Sciences University of Hawai'i at Mānoa Honolulu Hawaii USA

## Abstract

Compound‐specific isotope analyses (CSIA) and multivariate “isotope fingerprinting” track biosynthetic sources and reveal trophic interactions in food webs. However, CSIA have not been widely applied in the study of marine symbioses. Here, we exposed a reef coral (*Montipora capitata*) in symbiosis with Symbiodiniaceae algae to experimental treatments (autotrophy, mixotrophy, heterotrophy) to test for trophic shifts and amino acid (AA) sources using paired bulk (δ^13^C, δ^15^N) and AA‐CSIA (δ^13^C_AA_, δ^15^N_AA_). Treatments did not influence carbon or nitrogen trophic proxies, thereby not supporting nutritional plasticity. Instead, hosts and symbionts consistently overlapped in essential‐ and nonessential‐δ^13^C_AA_ (11 of 13 amino acids) and trophic‐ and source‐δ^15^N_AA_ values (9 of 13 amino acids). Host and symbiont trophic‐δ^15^N_AA_ values positively correlated with a plankton end‐member, indicative of trophic connections and dietary sources for trophic‐AA nitrogen. However, mass balance of AA‐trophic positions (TP_Glx–Phe_) revealed heterotrophic influences to be highly variable (1–41% heterotrophy). Linear discriminant analysis using *M. capitata* mean‐normalized essential‐δ^13^C_AA_ with previously published values (*Pocillopora meandrina*) showed similar nutrition isotope fingerprints (Symbiodiniaceae vs. plankton) but revealed species‐specific trophic strategies. *Montipora capitata* and Symbiodiniaceae shared identical AA‐fingerprints, whereas *P. meandrina* was assigned to either symbiont or plankton nutrition. Thus, *M. capitata* was 100% reliant on symbionts for essential‐δ^13^C_AA_ and demonstrated autotrophic fidelity and contrasts with trophic plasticity reported in *P. meandrina*. While *M. capitata* AA may originate from host and/or symbiont biosynthesis, AA carbon is Symbiodiniaceae‐derived. Together, AA‐CSIA/isotope fingerprinting advances the study of coral trophic plasticity and are powerful tools in the study of marine symbioses.

Scleractinian reef corals are mixotrophic cnidarians that form a mutualistic symbiosis with Symbiodiniaceae microalgae (formerly *Symbiodinium*) (LaJeunesse et al. 2018). The success of the coral‐Symbiodiniaceae symbiosis in nutrient‐poor tropical and subtropical seas is underpinned by nutritional exchanges. Symbiont photosynthesis supports coral metabolism and calcification (Muscatine et al. 1981) by providing the host with low‐molecular‐weight compounds (i.e., glucose, organic and amino acids), glycoconjugates, and free fatty acids (Trench 1971*b*; Markell and Trench 1993; Papina et al. 2003; Whitehead and Douglas 2003), as well as additional compounds from photosynthetic‐dependent dissolved inorganic nitrogen (DIN) assimilation and the uptake of dissolved free amino acids (DFAAs) (Wang and Douglas 1998; Kopp et al. 2013). In return, the coral supplies its endosymbionts with chemical building blocks derived from host metabolism (i.e., nitrogenous waste, metabolic inorganic carbon) (Wang and Douglas 1998). Supplemental to Symbiodiniaceae nutrition (collectively, autotrophy), reef corals exploit a variety of water column resources including dissolved organic compounds, bacteria, detritus, and a range of phytoplankton and zooplankton to meet energetic needs (collectively, heterotrophy) (Houlbrèque and Ferrier‐Pagès 2009).

Heterotrophic sources of nutrition are vital to reef corals, as symbiont‐derived autotrophic products are low in nitrogen and phosphorus (Falkowski et al. 1984). In healthy individuals, prey capture may account for 15–50% of energy demand (Houlbrèque and Ferrier‐Pagès 2009); although, host and endosymbiont assimilation of dissolved organic (i.e., DFAA, urea) and inorganic nitrogen (i.e., ${\mathrm{NO}}_{3}^{-}$, ${\mathrm{NH}}_{4}^{+}$) may fully sustain coral nitrogen budgets (Grover et al. 2008). The relative contribution of heterotrophy to the diet of corals can vary according to coral species (Palardy et al. 2005), environmental conditions (Fox et al. 2018), and physiological states (Grottoli et al. 2006). Corals are under mounting threats from local and global stressors, and trophic plasticity contributes to coral physiological resilience under environmental change (Grottoli et al. 2006). Therefore, testing the causes and consequences of nutritional plasticity in corals is of substantial interest.

Stable isotope analyses have been widely applied to study organismal physiology, trophic ecology, and biogeochemical cycles in terrestrial and marine ecosystems. In particular, variations in carbon and nitrogen isotopic composition have been used to determine the trophic ecology of reef corals across spatiotemporal scales (Alamaru et al. 2009) and in response to physiological stress (Baumann et al. 2014) or discrete Symbiodiniaceae associations (Baker et al. 2018; Wall et al. 2020). In some corals, greater reliance on heterotrophic feeding can be driven by light attenuation across depths (Alamaru et al. 2009), seawater turbidity (Anthony and Fabricius 2000), or in regions with high or variable oceanic productivity (Fox et al. 2018). In addition, heterotrophy may increase in some corals in response to disruption of the coral‐Symbiodiniaceae symbiosis during bleaching (Grottoli et al. 2006). Carbon isotope values are most often applied to corals as a means of identifying greater contributions of ^13^C‐depleted heterotrophic prey relative to ^13^C‐enriched autotrophic photosynthates (Muscatine et al. 1989; Laws et al. 1997). Based on these principles, more negative δ^13^C values in the host, or host values relative to those measured in the symbionts (i.e., host—symbiont δ^13^C values [i.e., δ^13^C_H–S_]), have been used to indicate a greater reliance on heterotrophic nutrition (Rodrigues and Grottoli 2006).

Early pioneering studies showed species‐specific isotope values in corals in low‐light, deep habitats, with lower δ^13^C and higher δ^15^N values interpreted as greater reliance on heterotrophy (Muscatine et al. 1989; Muscatine and Kaplan 1994). However, there are important considerations and limitations to isotope‐based inferences on animal diets, especially in the study of symbioses. For instance, changes in tissue composition (i.e., ^13^C‐depleted tissue lipids, protein : lipid : saccharide ratio) (Cooper et al. 2011; Wall et al. 2019), photosynthesis : respiration ratio (Swart et al. 2005), and shifts in symbiont communities (Wall et al. 2020) can all coincide with changes in depth, with each also influencing tissue δ^13^C values. In the case of nitrogen, spatial and temporal variability in nitrogen sources at the base of the food web can lead to different isotope values in consumers despite similar trophic levels (Heikoop et al. 2000). Trophic enrichment factors observed in conventional predator–prey interactions are also attenuated in corals due to internal nitrogen cycling between host and symbiont (Reynaud et al. 2009). Therefore, metabolic effects, tissue composition and turnover rates, and different isotopic compositions of source compounds at the base of the food web have each contributed to the uncertainty in deciphering flexibility from fidelity in coral trophic strategies.

Fundamental questions remain in our understanding of the mechanisms governing patterns of stable isotope values in the coral‐Symbiodiniaceae symbiosis. Compound‐specific isotope analyses (CSIA) of individual macromolecules, such as amino acids and fatty acids (FAs) (*see* review by Ferrier‐Pagès and Leal 2019), may be useful in disentangling biological changes in an organism's nutrition (or metabolism) from unconstrained variance in the form of fractionation‐mediated effects. Accordingly, CSIA may clarify central processes within the coral holobiont (i.e., nutrient cycling) and illuminate trophic interactions previously obscured in the study of mixotrophic symbioses. While bulk tissue isotope analyses represent a collective average of all macromolecules in a sample, CSIA use molecular approaches based on established metabolic pathways and their isotopic discrimination through trophic transfer and consumer biochemistry (McMahon and Newsome 2019). For instance, most animals are incapable of synthesizing all amino acids *de novo*; therefore, certain amino acids propagate through food webs without isotope fractionation and are effective tracers of consumer diets (Ohkouchi et al. 2017; McMahon and Newsome 2019). CSIA also have greater resolution and can simultaneously provide information on dietary food sources, nutrition, and physiology of an organism (Ohkouchi et al. 2017; Whiteman et al. 2019). Recently, carbon AA‐CSIA (Fox et al. 2019), nitrogen AA‐CSIA (Fujii et al. 2020; Martinez et al. 2020), and FA‐CSIA (Teece et al. 2011) have been applied in corals to examine nutritional modes at biological (i.e., within and among species) and environmental levels (i.e., shallow and mesophotic habitats). However, to date, few CSIA of reef corals exist and AA‐CSIA have been limited to ecological studies without experimental manipulation of feeding in controlled laboratory conditions.

Here we use bulk isotope and AA‐CSIA of carbon and nitrogen to test for trophic plasticity in the reef‐building coral *Montipora capitata* (Dana, 1846) exposed to manipulative nutrition treatments (light‐by‐feeding treatments). While the influence of light and food availability on bulk tissue isotope values in corals has been extensively studied, this work represents the first tandem analysis of δ^13^C_AA_ and δ^15^N_AA_ values for corals and Symbiodiniaceae (and a heterotrophic food source) under manipulative nutrition regimes. We assessed changes in individual δ^13^C_AA_ and δ^15^N_AA_ values and the dynamic patterns of multivariate amino acid data. Multivariate analyses of essential‐δ^13^C_AA_, termed essential amino acid “fingerprints,” have previously been used to identify origins of amino acids across food webs and primary producers (Larsen et al. 2009). Multivariate amino acid fingerprinting reflects an organism's capacity for amino acid biosynthesis and not the isotope ratio of the sample, which can vary due to local environmental conditions. Therefore, AA‐CSIA fingerprinting data can incorporate a wide variety of samples across a range of geographic locations or environmental settings to determine nutritional sources. Considering few AA‐CSIA data exist for coral and Symbiodiniaceae, we examined isotope values at several scales to determine sources of variation while limiting a priori assumptions. Using individual δ^13^C_AA_ and δ^15^N_AA_ values and isotope fingerprints consisting of 13 amino acids carbon (essential *and* nonessential) and nitrogen (trophic *and* source), we hypothesized that coral autotrophy under ecologically relevant light treatments would result in convergence in coral‐Symbiodiniaceae amino acid values and isotope fingerprints. Second, we hypothesized that heterotrophy treatments would cause divergence in coral‐Symbiodiniaceae amino acid values in favor of greater overlap with isotope values of the plankton community, and this effect would be most pronounced in the coral host. Finally, we combined our data with other published examples of essential‐δ^13^C_AA_ values in corals to examine patterns in coral‐Symbiodiniaceae and heterotrophic food sources to test for species‐specific patterns of trophic plasticity and amino acid sources within a single analytical framework.

## Materials and methods

### Coral collection

On 05 December 2017, two colonies (20 cm diameter) of *M. capitata* were collected at <1 m depth from The Point Reef at Moku o Lo'e (Coconut Island) at the Hawai'i Institute of Marine Biology (HIMB) (21°25′59.6″N, 157°47′11.7″W) on the windward side of O'ahu, Hawai'i. Colonies were spatially separated (5 m from each other) and at different perpendicular distances to the edge of the reef crest (ca. 5 vs. 10 m). While symbiont communities were not assessed, we chose colonies visually identified as “brown” and “orange” color morphs, which are common in Kāne'ohe Bay and are known to harbor different symbiont communities (Wall et al. 2020). Colonies were fragmented into nine ramets of approximately equal size (ca. 10 cm height) and placed into three flow‐through aquaria (50 liter) at a density of three ramets from each coral genet per treatment (*n* = 2 genets treatment^−1^). Aquaria received filtered seawater (1.0 *μ*m) from Kāne'ohe Bay maintained at ambient conditions (ca. 25°C) using in‐tank heaters, chilled seawater, and a real‐time temperature control system (Apex Controllers, Neptune Systems); flow was provided by a submersible aquarium pump. Photosynthetically active radiation (PAR) conditions were provided by light emitting diode lamps programmed on a 12L : 12D cycle and a diel ramp. Irradiances increased from 07:00 to 11:30 hrs to peak irradiances of 475 *μ*mol photons m^−2^ s^−1^, and decreasing from 14:30 to 19:00 hrs; daily integrated light intensities were 12 mol photons m^−2^ d^−1^. All corals were allowed to acclimate to tank conditions for 7 d before the start of light and feeding treatments (detailed below). During the acclimation period, corals were not fed supplemental heterotrophic prey.

### Experimental treatments

Corals were exposed to three experimental nutrition treatments in the aquaria used in post‐collection acclimation: light and no heterotrophic feeding (Light‐Not Fed [L‐NF]), light and heterotrophic feeding (Light‐Fed [L‐F]), and darkness with heterotrophic feeding (Dark‐Fed [D‐F]). Treatments were defined as interactions of combined light‐by‐feeding conditions to produce treatments based on ratios of autotrophy to heterotrophy: 1 : 0 (L‐NF, autotrophic), 1 : 1 (L‐F, mixotrophic), and 0 : 1 (D‐F, heterotrophic). Although *M. capitata* is not found in complete darkness in nature, it has the capacity to survive and persist under extremely low‐light conditions. *Montipora capitata* is commonly found at water depths of 40–100 m in the 'Au'au Channel separating the Hawaiian Islands of Maui and Lana'i where PAR is about 1% of surface levels (Pyle et al. 2016). This species also survives under extremely low‐light conditions in shallow habitats of Kāne'ohe Bay where surface light is attenuated by 86% at 8 m depth (Wall et al. 2020). While the D‐F treatment has limited ecological relevance, this treatment was designed to bring a cessation to Symbiodiniaceae photosynthesis during the experimental period.

Corals in feeding treatments were provided with plankton freshly collected using a plankton tow (63‐*μ*m mesh) off the leeward side of HIMB and size‐fractioned to remove debris greater than 250 *μ*m. Microscopy showed this plankton size fraction (63–250 *μ*m) to be dominated by copepods, zoea, and gastropod larvae. A concentrated plankton sample collected on 19 January 2018 (63–250 *μ*m) was saved as a heterotrophic food source isotopic end‐member. Plankton was provided to corals in feeding treatments thrice weekly for 3 h during midday (13 : 00–16 : 00 h). Previous work has demonstrated plankton and POM samples from seawater adjacent to patch reefs in Kāne'ohe Bay show limited spatial and seasonally variability in δ^13^C and δ^15^N values and low δ^15^N variability (~ 2‰) due to size fractions (Wall et al. 2020) (Supporting Information Table S1). Therefore, it is reasonable to expect isotope values in the plankton samples of a consistent size fraction were relatively stable during our short experimental time period. Concentrated tow materials were added directly into treatment tanks while turning off the supply of seawater entering tanks to avoid prey escape; final plankton concentration (mean ± SE) in each tank was 1630 ± 322 plankton L^−1^. Unfed corals experienced the same environmental conditions as fed corals during feeding (i.e., periodic cessation of flow‐through seawater). Prey ingestion rates for *M. capitata* were not quantified, as the goal of the feeding treatments was to give corals *ad libitum* access to plankton (Edmunds 2011) to test the influence of heterotrophy on tissue isotope values. *Montipora capitata* feeding has been previously shown to range from 5 to 32 plankton per gram of coral ash‐free dry weight biomass per hour in bleached and pigmented colonies (Grottoli et al. 2006). Corals were exposed to treatments for 29 d (14 December 2017–11 January 2018) and immediately frozen at −80°C until further processing.

### Tissue stable isotope analysis

Coral tissues were removed from the skeleton using an airbrush filled with double‐distilled water (ddH_2_O) and attached to a compressed air cylinder. To obtain enough tissue for AA‐CSIA, the three replicate coral ramets within each treatment tank were pooled to produce a single coral blastate for each genet within a treatment (*n* = 2 genets per treatment). The isolated tissue blastate was kept on ice, briefly homogenized, and filtered through 20‐*μ*m mesh to remove skeletal debris (Wall et al. 2020). Host and symbiont tissues were separated by repeated centrifugation and ddH_2_O rinses (Muscatine et al. 1989), and isolated tissues were lyophilized and stored at room temperature until analyzed. Isotopic values are reported in delta values (*δ*) using per mill (‰) notation relative to standard materials (Vienna Pee‐Dee Belemnite [V‐PDB] and atmospheric N_2_ standards [Air] for carbon and nitrogen, respectively). Samples for bulk tissue carbon (δ^13^C) and nitrogen (δ^15^N) isotope analyses and C : N ratios for coral host, symbiont algae and plankton tissues (ca. 1 mg) were packed in tin capsules and measured on a Costech elemental combustion system coupled to a Thermo‐Finnigan Delta Plus XP isotope ratio mass spectrometer. Sample analytical accuracy and precision (δ^13^C and δ^15^N) was <0.2‰ (*see* Supporting Information Appendix S1).

### Individual amino acid isotope analyses

Isotopic analysis of amino acids in all samples (coral host, symbiont algae, plankton) was performed by subjecting tissue samples to acid hydrolysis, carboxyl terminus esterification, and amine group trifluoroacetylation (Hannides et al. 2013; Shih et al. 2020) (*see* Supporting Information Appendix S1). Acid hydrolysis was performed by heating (150°C) approximately 15 mg of tissue in 6 N HCl, evaporating to dryness, redissolving hydrolysate in 0.01 N HCl, filtering (0.2‐*μ*m polyethersulfone filter), purifying by cation exchange (Dowex 50WX8‐400), and amino acid elution with ammonium hydroxide. Hydrolyzed tissues were esterified and the amine group was trifluoroacetylated. Finally, solvent extraction in P‐buffer (KH_2_PO_4_ + Na_2_HPO_4_ in milli‐Q water, pH 7) was used to further purify samples (Shih et al. 2020). Chloroform was used to partition acylated amino acids, and following solvent evaporation sample trifluoracetylation was repeated to maximize derivitization. Samples were stored frozen in 3 : 1 methylene chloride : TFAA at −20°C until analyzed.

At the time of analysis, samples were evaporated under N_2_ at room temperature and redissolved in 50–100 *μ*L ethyl acetate. Carbon stable isotope composition of amino acids was determined using a Thermo Scientific MAT 253 mass spectrometer coupled to a Thermo Scientific Trace GC Ultra gas chromatograph via a Thermo Scientific Conflo IV (*see* Arthur et al. 2014). For δ^13^C determination, internal reference compounds of known isotopic composition—aminoadipic acid (AAA) and norleucine (Nor) and underivitized *n*‐C_20_ alkane—were co‐injected with samples and used to determine accuracy and precision. Between triplicate runs of each sample a suite of amino acids of known isotopic composition that were prepared alongside the samples were analyzed; the AAA, Nor, and *n*‐C_20_ were also co‐injected with these amino acids. For isotopic correction of unknown amino acids, an average correction factor was derived from the amino acid suites run before and after the triplicate sample analysis and applied to measured isotope ratios (*see* details in Arthur et al. 2014). All amino acids were analyzed in triplicate and isotopic values are reported in δ‐notation relative to V‐PDB.

Nitrogen stable isotope composition of amino acids was determined using a Delta V Plus mass spectrometer interfaced with a Trace GC gas chromatograph through a GC‐C III combustion furnace (980°C), reduction furnace (650°C), and liquid nitrogen cold trap (*see* details in Hannides et al. 2013). All samples were analyzed in at least triplicate. Internal reference compounds (AAA and Nor) were co‐injected with samples and served as a normalizer for sample amino acids δ^15^N values and an internal tool for monitoring combustion reactor degradation and sample injection accuracy. Measurement accuracy was determined using the known δ^15^N value of aminoadipic acid to determine the measured δ^15^N value of norleucine, and vice versa. In addition, a full amino acid reference suite (15 amino acids) of known isotopic composition was also analyzed before and after each three sample measurements. A process blank (subject to the same hydrolysis and derivatization steps) was analyzed in the same manner as samples and did not contain detectable amino acids.

### Amino acid classification

The classification of amino acids using carbon isotope values is often grouped as acquired through diet (i.e., essential) and synthesized *de novo* from cellular carbon pools (i.e., nonessential). Essential amino acids include isoleucine (Ile), leucine (Leu), lysine (Lys), methionine (Met), phenylalanine (Phe), threonine (Thr), and valine (Val). The intact carbon skeletons of essential amino acids are synthesized in a variety of pathways by bacteria and primary producers. Consumers acquire essential amino acids through dietary proteins and incorporated them into tissues expressing little isotope fractionation. Conversely, the nonessential amino acids glycine (Gly), serine (Ser), alanine (Ala), glutamic acid (Glx), aspartate (Asp), proline (Pro), arginine (Arg), and tyrosine (Tyr) are synthesized from carbon pools or direct routing of nonessential amino acids sourced from dietary protein (*see* McMahon and Newsome 2019).

Amino acid nitrogen isotope values are controlled by the enzymatic processes of transamination and deamination, which lead to isotope fractionation (e.g., O'Connell 2017). Protein δ^15^N values for amino acids are classified as “source” and “trophic” amino acids (sensu Popp et al. 2007) and are based on empirical studies of nitrogen incorporation into amino acids. Source amino acids, including Phe, Met, Lys, show minimal C—N bond breakage in their metabolism and discriminate little between diet and consumer and are thought to represent baseline nitrogen values in the absence of trophic discrimination. In contrast, the trophic amino acids, including Glx, Asp, Ala, Ile, Leu, Pro, and Val, undergo extensive transamination and deamination; thus, their δ^15^N values increase with each trophic transfer (Ohkouchi et al. 2017).

The essential (EAA) and nonessential (NEAA) and trophic and source amino acids have been previously applied in birds, mammals, and invertebrates (*see* Whiteman et al. 2019). However, corals can synthesize some amino acids *de novo* and translocation of amino acids between symbiont partners has obscured origins of amino acid synthesis (Trench 1971*b*; Fitzgerald and Szmant 1997; Wang and Douglas 1999); therefore, applying these bins to corals should be considered with caution. Due to this uncertainty, we follow the previously published examples of essential and nonessential amino acid grouping for carbon isotope analysis of corals (Fox et al. 2019) and other animals (Whiteman et al. 2019). For trophic and source amino acids, we used amino acid designation outlined in Whiteman et al. (2019) and include those amino acids where information is limited, unknown, or where behavior is “source‐like” (i.e., glycine, serine) as source‐amino acids.

### Trophic position and trophic proxies

Trophic position was calculated as the difference between δ^15^N values of glutamic acid (glutamate, Glx) and phenylalanine, following (Chikaraishi et al. 2009):

(1) $$\text{Trophic position}\;\left({\mathrm{TP}}_{\mathrm{Glx}–\mathrm{Phe}}\right)=\left(\left(\left(\delta^{15}N_{\mathrm{Glx}}–\delta^{15}N_{\mathrm{Phe}}\right)–3.4\right)/7.6\right)+1,$$

where an assumed *β* value of 3.4‰ for differences in δ^15^N values of glutamic acid and phenylalanine in primary producers and a 7.6‰ enrichment (Δ values) in ^15^N between glutamic acid and phenylalanine at each trophic transfer. Fractionation factors for δ^15^N_AA_ in prokaryotic and eukaryotic phytoplankton cultures and isolated Symbiodiniaceae are similar and support both the grouping of amino acids in these taxa as “fractionating” and “nonfractionating” groups and the application of TP_Glx–Phe_ calculations (McCarthy et al. 2013; Fujii et al. 2020) (*see* Supporting Information Appendix S1 for details on testing canonical *β* and Δ values). Uncertainty in trophic position was determined using propagation of errors (Jarman et al. 2017). To estimate fractional contributions of heterotrophy to coral nutrition, we used trophic positions calculated from δ^15^N_AA_ values of glutamic acid and phenylalanine and a simple linear two‐component mixing model that yields a propagated uncertainty (Phillips and Gregg 2001). This approach uses a first‐order Taylor series approximation to estimate the propagated variance in the contribution of each source.

An additional proxy for trophic position that does not rely on assumed values of *β* or Δ, the difference in the δ^15^N‐weighted mean values for trophic and source amino acids (Bradley et al. 2015), was also calculated using trophic (alanine, leucine, glutamic acid) and source amino acids (lysine, phenylalanine), following (Hayes et al. 1990):

(2) $$\delta^{15}N_{{\bar{x}}_{w}}=\frac{\sum \frac{\delta^{15}N_{x}}{\sigma_{x}^{2}}}{\sum \frac{1}{\sigma_{x}^{2}}},$$

where δ^15^N_*x*_ is the nitrogen isotopic value of a specified amino acid within the trophic or source category, and *σ*_*x*_ is the standard deviation of the amino acid based on triplicate analysis (Shih et al. 2020).

### Summed variance in trophic amino acid δ^15^N (∑*V*)

The summed variance (∑*V*) index was applied to trophic‐δ^15^N_AA_ values as a metric for heterotrophic resynthesis of organic matter by bacteria (McCarthy et al. 2007), which is a potential supplemental source of Symbiodiniaceae nutrition. The sum of the δ^15^N_AA_ variance among individual trophic amino acids (alanine, leucine, proline, aspartic acid, and glutamic acid) was used to calculate ∑*V* for host and symbiont tissues according to experimental treatment.

### Data analyses

Amino acid carbon and nitrogen isotope values in the coral and Symbiodiniaceae were examined in a permutational multivariate ANOVA (PERMANOVA) using Euclidean distance matrices to test for differences in tissue fraction (host or symbiont) and nutrition treatments in the package *vegan* (Oksanen et al. 2019). In order to meet requirements for PERMANOVA, amino acid isotope values were analyzed as absolute values (for carbon) or with a constant applied (in case of nitrogen isotope values). Principal components analyses (PCA) of a scaled and centered correlation matrix were then applied to amino acid carbon and nitrogen isotope values. Correlation vectors were plotted on PC‐biplots for amino acids showing significant correlations with PCs. Analyses were grouped at the level of (1) tissue fraction and (2) treatment to visualize the relationship between coral‐Symbiodiniaceae amino acid isotope values and those observed in the pooled plankton sample (i.e., the heterotrophic food source). Carbon and nitrogen isotope values for corals and Symbiodiniaceae bulk tissue, individual amino acids, and indices (e.g., trophic position, δ^15^N‐weighted mean, ∑*V*) were analyzed using a linear model with tissue fraction (host or symbiont) and nutrition treatments as fixed effects.

To compare the multivariate essential‐δ^13^C_AA_ fingerprints from *M. capitata* from Kāne'ohe Bay (this study) with those of the only other example of δ^13^C_AA_ values in reef corals and Symbiodiniaceae (Palmyra *Pocillopora meandrina*) in relation to allochthonous food sources (plankton and particulate organic carbon [POM]) (Fox et al. 2019), we used a linear discriminant analysis (LDA). The LDA included δ^13^C values of six essential amino acids (isoleucine, leucine, lysine, phenylalanine, threonine, valine) in hosts, symbionts, plankton, and POM. Isotope values were normalized to their sample mean to allow for comparison of essential‐δ^13^C_AA_ patterns among groups (Larsen et al. 2009). Using mean‐normalized essential‐δ^13^C_AA_, fingerprinting has been applied in other systems to trace sources of carbon through terrestrial and aquatic food webs (Larsen et al. 2013) and removes baseline spatiotemporal variability in δ^13^C values (Larsen et al. 2020). Using nutrition sources (symbionts, plankton‐POM), we obtained predictions of group membership from leave‐one‐out cross‐validation using the function *lda* in the package MASS (Venables and Ripley 2002). Amino acid coefficients contributing most to class selection were obtained and the *predict* function was used to assign coral host membership to nutrition groups for both *M. capitata* and *P. meandrina*. Linear discriminant axes (LD1, LD2) were plotted with ellipses (95% standard deviation of group mean) for nutrition sources. Raw essential‐δ^13^C_AA_ values in Palmyra plankton and POM are not distinct (Fox et al. 2019), therefore ellipses were drawn according to autotrophic (symbiont‐derived) or heterotrophic (plankton + POM) nutrition sources.

To supplement the LDA from Hawai'i and Palmyra, we subsequently applied the same LDA approach above using values of the coral holobiont (*Acropora haraonis* + Symbiodiniaceae) and plankton (> 333 *μ*m) from the Red Sea (McMahon et al. 2015) using mean‐normalized essential‐δ^13^C_AA_ values for five essential amino acids (isoleucine, leucine, phenylalanine, threonine, valine [lysine not available]). This analysis offers a comparison of hosts, symbionts, holobionts, and planktonic food sources across three locations and provides insight into the sources of coral nutrition and comparability of planktonic food sources among coral reefs.

Finally, we sought to test (1) whether Symbiodiniaceae show different essential‐δ^13^C_AA_ fingerprints compared to diverse groups of cultured and free‐living plankton and (2) how similar essential‐δ^13^C_AA_ fingerprints are in diverse plankton assemblages. To accomplish this, we surveyed the literature and compiled values of the five essential‐δ^13^C_AA_ for plankton, POM, and microalgae using examples from Kāne'ohe Bay (O'ahu, Hawai'i, this study), Station Aloha (open ocean, north of O'ahu, Hannides et al. 2013), Palmyra (Fox et al. 2019), the Red Sea (McMahon et al. 2015), and in cultured microalgae (grouped chlorophytes, chrysophytes, cyanobacteria, diatoms, and haptophytes; Larsen et al. 2013). Pooling the cultured microalgae was previously justified using mean‐normalized essential‐δ^13^C_AA_ values due to limited differences among groups (Larsen et al. 2013). Mean‐normalized essential‐δ^13^C_AA_ values in these free‐living/cultured plankton, which represent a range in heterotrophic food sources, were compared to those of Symbiodiniaceae isolated in corals from Hawai'i and Palmyra using PCA of a scaled and centered correlation matrix.

All statistical analyses were performed in R version 3.6.1 (R Core Team 2019). Data and code to reproduce analyses and figures are available at GitHub (https://github.com/cbwall/CSIA-corals) and archived at Zenodo (http://doi.org/10.5281/zenodo.4527785).

## Results

### Bulk tissue isotope analyses

**Fig. 1:**
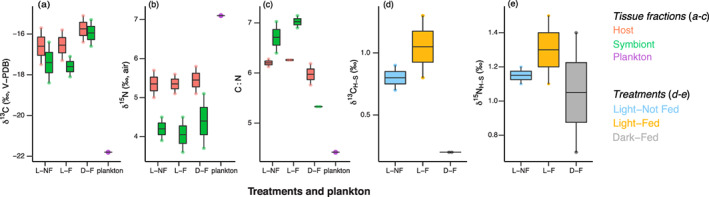
(**a**–**e**) Bulk tissue δ^13^C and δ^15^N values and C : N for coral hosts and Symbiodiniaceae symbionts and their relative differences (δ^13^C_H–S_, δ^15^N_H–S_) exposed to three Light‐by‐Feeding nutrition treatments and their relation to a pooled plankton sample. Treatments are L–NF (Light–Not Fed, autotrophic), L–F (Light–Fed, mixotrophic), D–F (Dark–Fed, heterotrophic). Boxplots are *n =* 2, except for plankton (*n* = 1).

Carbon isotope values did not differ between host and symbiont tissue fractions or in response to treatments (*p* ≥ 0.190) (Supporting Information Table S2, Fig. 1a). Nitrogen isotope values were higher in the host than the symbiont (*p* = 0.005) (Fig. 1b) but were not affected by treatments (*p =* 0.831). Mean C : N ranged from 5.3 to 7.0 and did not differ between host and symbiont tissues (*p* = 0.420). Tissue C : N was lower in the D‐F treatment compared to others (*p* = 0.025), with symbiont C : N declining and approaching those in plankton (C : N = 4.4) (Fig. 1c). The difference in isotope values in the host relative to the symbiont for carbon (*p* = 0.064) and nitrogen was not affected by treatments (*p =* 0.769) (Fig. 1d,e), although δ^13^C_H–S_ tended to be 0.5–1‰ lower in the D‐F treatment relative to others. Overall, the carbon and nitrogen isotope values of the pooled plankton sample were lower (δ^13^C) and higher (δ^15^N) than those values observed in both the coral host and Symbiodiniaceae symbionts (Fig. 1).

### Amino acid isotope analyses

Thirteen amino acids were extracted from coral tissue, Symbiodiniaceae, and a pooled plankton end‐member (Supporting Information Table S3). Mean coral host δ^13^C_AA_ values ranged from −24.3 to −8.1‰ and were generally higher than those observed in the symbionts (9 of 13 amino acids) but differences in host and symbiont δ^13^C_AA_ values ranged from −0.8 to +3.8‰. Carbon isotope values in plankton amino acids were on average 5.5 and 4.7‰ lower than those in coral host and Symbiodiniaceae, respectively (Supporting Information Table S3). Coral δ^15^N_AA_ values ranged from −1.1 to +7.0‰ but were variable in relation to δ^15^N_AA_ values in the symbiont. Individual coral δ^15^N_AA_ values ranging from −2.3 to +2.7‰ relative to Symbiodiniaceae, with glycine, threonine, and tyrosine in particular having higher δ^15^N values in the symbionts. δ^15^N_AA_ values in the plankton sample were on average 2.5 ‰ higher relative to corals and Symbiodiniaceae.

PERMANOVA results showed the multivariate carbon isotope fingerprint (i.e., δ^13^C_AA_) of 13 amino acids did not differ between corals and their symbionts (*p* = 0.056), nutrition treatments (*p* = 0.470), or their interaction (*p* = 0.771) (Table 1). The multivariate nitrogen isotope fingerprint (i.e., δ^15^N_AA_), however, did differ between host and symbiont tissue fractions (*p* = 0.017), but was not affected by treatments (*p* = 0.911) or the fraction × treatment interaction (*p* = 0.599) (Table 1).

**Table 1:** Results of PERMANOVA testing effects of tissue fraction and nutrition treatment on amino acid carbon and nitrogen isotope values.*

*Factor*	df	SS	*R* ^2^	*F*	*p*
Amino acid carbon isotope values					
Fraction	1	6.931	0.153	1.724	0.056
Treatment	2	7.895	0.174	0.982	0.470
Fraction × treatment	2	6.369	0.141	0.792	0.771
Residual	6	24.128	0.532		
Total	11	45.323	1.000		
Amino acid nitrogen isotope values					
Fraction	1	7.065	0.182	2.042	**0.017**
Treatment	2	4.753	0.122	0.687	0.911
Fraction × treatment	2	6.315	0.162	0.912	0.599
Residual	6	20.763	0.534		
Total	11	38.896	1.000		

df, degrees of freedom; SS, sum of squares.

^*^ “Fraction” is host coral or symbiont Symbiodiniaceae tissue. “Treatment” represents combination of Light‐by‐Feeding nutrition treatments: Light–Not Fed, Light–Fed, Dark–Fed. Bolded *p‐*value represents significant effects (*p* < 0.05).

**Fig. 2:**
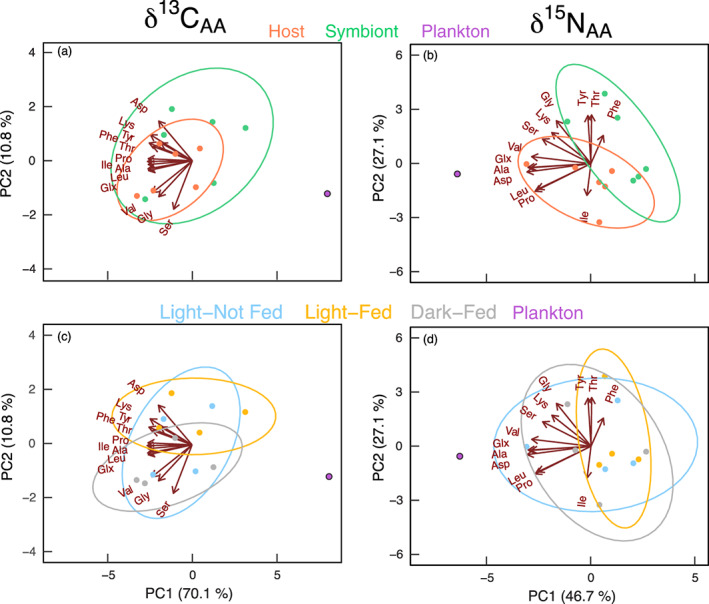
(**a**–**d**) Principal component analyses of δ^13^C (*left*) and δ^15^N (*right*) values of individual amino acids in coral hosts, Symbiodiniaceae symbionts, and a pooled plankton sample in relation to tissue fractions (**a**,**b**) and treatments (**c**,**d**). Ellipses represent 90% standard deviation with arrows for individual amino acids being significant (*p* < 0.05) correlation vectors.

PCA showed two PCs explained 81% (carbon) and 74% (nitrogen) of variance in amino acid isotope values in the coral, Symbiodiniaceae, and pooled plankton sample (Fig. 2). The PCA ellipses followed PERMANOVA results, with host and symbiont amino acids overlapping for δ^13^C_AA_ values (Fig. 2a) but showing clear separation for δ^15^N_AA_ values (Fig. 2b). Treatment ellipses showed δ^13^C_AA_ values were most distinct between the L‐F and D‐F treatments (Fig. 2c). δ^15^N_AA_ ellipses overlapped but showed a more constrained distribution in the L‐F treatment (Fig. 2d). In relation to the plankton sample, vectors for individual amino acid δ^13^C values were negatively correlated with PC1, which had a positive correlation with the plankton sample. Therefore, carbon PCAs for tissue fraction and treatment effects on δ^13^C_AA_ values showed poor convergence or relation to those of the plankton (Fig. 2a,c). In contrast, amino acid vectors for δ^15^N_AA_ values in the host, symbionts, and the plankton were all negatively correlated with PC1. Six amino acid vectors (glutamic acid, alanine, aspartic acid, and valine) were particularly correlated with the plankton sample and also aligned more with host‐ellipses over those in the symbiont. In contrast, symbiont δ^15^N_AA_ ellipses and vectors positively correlated with PC2 were phenylalanine, tyrosine, and threonine (Fig. 2b). Treatments showed poor correlation with δ^15^N_AA_ values in the plankton, where ellipses for greater heterotrophy food availability (i.e., L‐F, D‐F) overlapped with those where heterotrophy was withheld (Fig. 2d).

### Carbon isotope values of individual amino acids

**Fig. 3:**
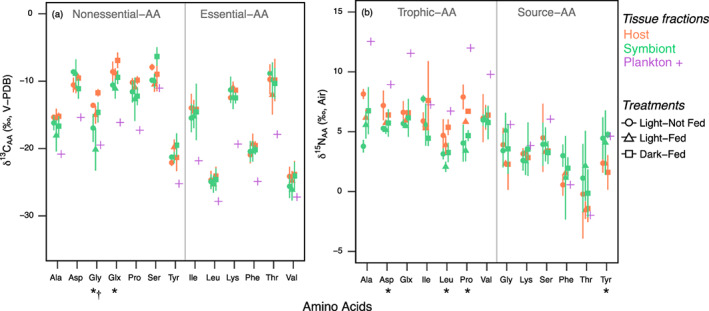
(**a**) δ^13^C and (**b**) δ^15^N values of individual amino acids in coral host, Symbiodiniaceae symbionts, in response to nutrition treatments relative to a pooled plankton sample. Treatments are Light–Not Fed (L–NF), Light–Fed (L–F), Dark–Fed (D–F); values are mean ± SD (*n* = 2), except for the plankton sample (*n* = 1). X‐axis *symbols* indicate significant differences (*p* < 0.05) between fractions (host and symbiont, *) and treatments (†).

For the 13 individual amino acids, carbon isotope values in host and symbiont fractions only differed for glycine (*p* = 0.004) and glutamic acid (*p* = 0.004) (Supporting Information Table S4), which were lower in symbiont tissues relative to the host (Fig. 3a). However, trends in δ^13^C_AA_ values were observed for alanine (*p =* 0.059) and proline (*p =* 0.055), which tended to also have lower isotope values in symbiont tissues (Supporting Information Fig. S1a). Nutrition treatments (i.e., light × feeding) showed limited effects on δ^13^C_AA_, with the exception of glycine (*p* = 0.016), which was significantly lower in L‐F relative to D‐F treatment (post hoc: *p* = 0.013) and lower in both the autotrophic and mixotrophic treatments (i.e., L–NF, L–F) (post hoc: *p* ≥ 0.172) (Fig. 3a, Supporting Information Fig. S1c). The remaining amino acids were not affected by treatments (*p* ≥ 0.076) (Supporting Information Table S4). Carbon isotope values in amino acids of the pooled plankton sample were generally lower for all amino acids measured in the host and symbiont; however, δ^13^C_AA_ values for valine, glycine, and serine overlapped with those observed in the plankton (Fig. 3a, Supporting Information S1a).

### Nitrogen isotope values of individual amino acids

Four amino acid nitrogen isotope values differed in host and symbiont fractions. δ^15^N_AA_ values were lower in symbiont fractions for leucine (*p* = 0.002), proline (*p* = 0.001), and aspartic acid (*p* = 0.036), but the isotope values of tyrosine were higher in the symbiont relative to the host (*p* = 0.017) (Supporting Information Table S5; Fig. 3b, Supporting Information Fig. S1b). δ^15^N_AA_ values did not differ between host and symbiont fractions for the other nine amino acids (*p* ≥ 0.103) (Supporting Information Table S5; Fig. 3, Supporting Information Fig. S1b). Nutrition treatments did not influence δ^15^N_AA_ values in any of the 13 amino acids, although a trend for lower δ^15^N_AA_ values in heterotrophy treatments (i.e., L‐F, D‐F) was observed for leucine (*p* = 0.069) (Fig. 3b, Supporting Information Fig. S1d). There was no significant difference in source amino acid δ^15^N values in the plankton, coral hosts, or symbionts, and trophic position calculations using glutamic acid and phenylalanine (e.g., Eq. 1, TP_Glx–Phe_) did not differ in host and symbiont fractions (*p* = 0.223) or in response to treatments (*p* = 0.998). Thus, source‐δ^15^N_AA_ values alone were ineffective in identifying sources of nutrition in *M. capitata*. Overall, the mean (± SD) host and symbiont TP_Glx–Phe_ ranged from 0.90 ± 0.08 to 1.35 ± 0.24, with the highest trophic positions measured in corals from treatments that were not fed; the plankton end‐member TP_Glx–Phe_ was 2.00 ± 0.22 (Supporting Information Table S6; Fig. S2a).

### Percent heterotrophy and δ^15^N_AA_ ∑*V*

Differences between plankton TP_Glx–Phe_ and those in coral host and symbionts were used to estimate fractional contributions of heterotrophy using TP_Glx–Phe_ in a mass balance two‐member mixing model that accounted for three end‐member trophic positions. First, the TP_Glx–Phe_ of Symbiodiniaceae primary producers is 1.0 (Supporting Information Table S6; but *see* also Martinez et al. 2020; Fujii et al. 2020); therefore, 100% autotrophic nutrition in the coral host would occur if the synthesis of glutamic acid by the coral host is entirely derived from translocation of symbiont‐derived nitrogen (amino or organic acids). Second, assuming the Δ_Glx–Phe_ value for heterotrophic feeding by coral hosts is similar to other heterotrophic organisms and zooplankton consumers (TP_Glx–Phe_ of 2.00; this study, Fujii et al. 2020), we anticipate corals feeding on 100% heterotrophy in the form of primary producers and algal detritus would have a TP_Glx–Phe_ of 2.0. These primary producers could be free‐living algae or *in hospite* Symbiodiniaceae, as corals can ingest phytoplankton (Leal et al. 2014) as well as digest symbionts (Titlyanov et al. 1996; Tanaka et al. 2018). Lastly, as a third end‐member, we consider a diet of zooplanktivory. The pooled plankton sample was a mix of microzooplankton and macrozooplankton at TP_Glx–Phe_ 2.00 ± 0.20. Therefore, we consider the host coral feeding heterotrophically on zooplankton to have a TP_Glx–Phe_ of 3.0, which is similar to a mean TP_Glx–Phe_ of 2.86 for a temperate heterotrophic octocoral (Grossowicz et al. 2020). Using the difference in TP_Glx–Phe_ between plankton, symbiont and host, the fractional contribution of heterotrophy to host nutrition ranged from (mean ± SD) 1% ± 9% to 41% ± 15%, being higher in mixing models assuming heterotrophy of TP_Glx–Phe_ 2.0 relative to 3.0 (Supporting Information Table S6). Surprisingly, these calculations resulted in higher % heterotrophy in the Light‐Not Fed treatment (21–41% heterotrophy) compared to treatments where corals were fed (1–6% heterotrophy) (Supporting Information Table S6).

Calculations of δ^15^N_AA_ ∑*V* (i.e., Sum‐V) did not differ among host or symbiont fractions (*p* = 0.088) or in response to treatments (*p* = 0.656) (Supporting Information Fig. S2b), and δ^15^N_AA_‐weighted means did not differ between the host and symbionts or between source and trophic amino acid (*p* ≥ 0.626) (Supporting Information Fig. S3).

### δ^13^C essential amino acids patterns among coral studies

**Fig. 4:**
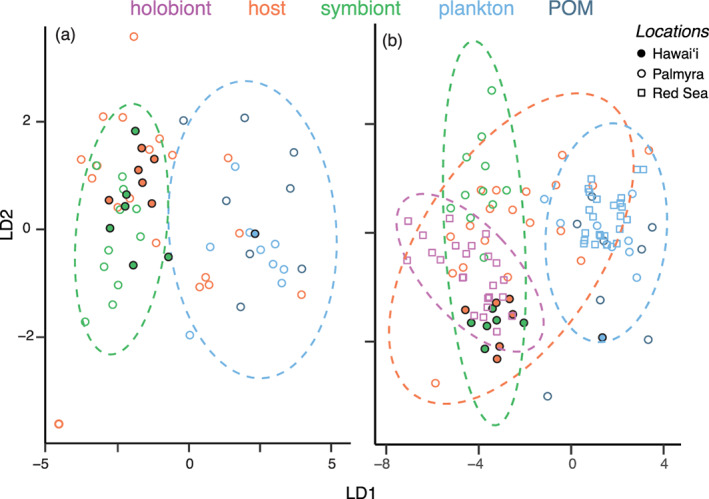
Linear discriminant analysis of mean‐normalized amino acid carbon isotope fingerprints using (**a**) six essential amino acids (isoleucine, leucine, lysine, phenylalanine, threonine, tyrosine, valine) and (**b**) five essential amino acids (without lysine). Ellipses represent 95% confidence ellipses for each nutrition group (autotrophy [symbiont] or heterotrophy [plankton‐POM]). In (**a**) and (**b**), Hawai'i data are host and symbiont (*n* = 6 [fragments from two reef genets in three experimental treatments]) and plankton (*n* = 1 [63–250 *μ*m]); Palmyra data are host (*n* = 19), symbionts (*n* = 11) (from 19 colonies [10 m]) and plankton (*n* = 9 [>163 *μ*m]) and POM (*n* = 8 [>0.7 *μ*m]) at four sites (Fox et al. 2019). Red Sea data in (**b**) are host+symbiont (holobionts, *n* = 23 colonies) and plankton (*n* = 23 [>333 *μ*m]) from eight sites (McMahon et al. 2015).

The δ^13^C values for amino acids from the pooled Kāne'ohe Bay, plankton sample (63–250 *μ*m) displayed similar patterns to those reported for plankton (> 163 *μ*m) and seawater POM (> 0.7 *μ*m) in Palmyra (Fox et al. 2019), although an offset was observed, with lower δ^13^C values for the Hawai'i plankton sample, particularly for nonessential amino acids (Supporting Information Fig. S4). Using mean‐normalized δ^13^C values for the six essential amino acids (i.e., δ^13^C_EAA_) of autotrophic and heterotrophic food sources from Hawai'i and Palmyra, linear discriminant analysis cross‐validation correctly identified class membership (71% overall), with 100% success rates for symbionts, but less so for plankton and POM (60% and 25%, respectively), which largely overlapped in their essential‐δ^13^C_AA_ values and are poorly differentiated nutritional sources (Fig. 4a). The first linear discriminant (LD1) explained 98% of group variation, and the essential amino acids contributing most to group separation along LD1 were valine (LD1 scaling: 0.64), leucine (0.55), isoleucine (−0.50), and phenylalanine (−0.34). The LDA predictions of coral host group membership showed the essential‐δ^13^C_AA_ fingerprint of all Hawai'i *M. capitata* corals to be autotrophic (i.e., grouped with symbionts), and *M. capitata* corals showed a high degree of overlap with symbionts in LD plots (Fig. 4). In contrast to *M. capitata*, Palmyra *P. meandrina* were more variable and plotted across autotrophic and heterotrophic food source groups (Fig. 4). The larger training data set of essential‐δ^13^C_AA_ values from Hawai'i and Palmyra assigned six of 19 *P. meandrina* corals to heterotrophic food sources, in agreement with group membership reported by Fox et al. (2019). Adding Red Sea coral and plankton samples to the mean‐normalized essential‐δ^13^C_AA_, LDA resulted in 83% effective group membership among food sources (symbionts, plankton, POM), with 94% and 100% of plankton and symbionts group membership being correctly assigned. Here, LD1 accounted for 99% of group variance. Six corals (all from Palmyra) were again assigned to heterotrophic food sources, whereas Hawai'i and Red Sea corals were all assigned to symbiont food sources (Fig. 4b).

PCA of mean‐normalized isotope values for five essential‐δ^13^C_AA_ of cultured microalgae and plankton/POM samples across five studies showed 62% of variance was explained by two PCs. Greater data dispersion were observed for microalgae relative to the plankton and POM; however, both groups overlapped (Supporting Information Fig. S5a). Adding Symbiodiniaceae symbionts into the data matrix increased variance explained (71% at two PCs). Symbionts were separated based on their location (Hawai'i, Palmyra) and/or host coral (*M. capitata, P. meadrina*), yet symbionts were distinct and did not overlap with microalgae, plankton, or POM sources (Supporting Information Fig. S5b).

## Discussion

Climate change and environmental stress threaten reef‐building corals; however, the inherent or flexible exploitation of heterotrophic nutrition can benefit corals experiencing abiotic stress and dysbiosis. Previous work using feeding assays (Grottoli et al. 2006) and isotope analyses of bulk tissues (Rodrigues and Grottoli 2006) and lipids (Baumann et al. 2014) has shown *M. capitata* grown in the laboratory utilizes heterotrophic nutrition to survive stressful events including bleaching. However, field data collected across environmental gradients (Wall et al. 2020) and during and after *in situ* bleaching events (Wall et al. 2019, but *see ex situ* bleaching Rodrigues and Grottoli 2006), do not support a tendency towards trophic plasticity in this species. Using both bulk and AA‐CSIA, we show minimal responses of *M. capitata* to nutrition treatments and do not observe this species to exploit opportunities for greater prey capture when resources are available, despite changes in autotrophic inputs. Results of a linear discriminant analysis of mean‐normalized essential‐δ^13^C_AA_ values showed autotrophic (symbiont) nutrition supported all *M. capitata* corals regardless of treatment. Treatments did drive greater variability in δ^15^N_H–S_ and declining δ^13^C_H–S_ for D‐F corals in particular, while also reducing C : N and glycine‐δ^15^N values. Across all treatments, host and symbiont trophic positions (i.e., TP_Glx–Phe_, difference in the δ^15^N‐weighted mean values for trophic and source amino acids) were lower than the plankton TP (~ 1 vs. plankton TP_Glx–Phe_ of 2), suggesting limited trophic connections to plankton‐derived nutrition compared to other cnidarian predators (Grossowicz et al. 2020). While treatments were not meant to produce changes in coral physiology at the scale of cellular bleaching, and results are not meant to be exhaustive in testing for heterotrophic plasticity at ecological scales, we nevertheless conclude healthy *M. capitata* exhibit low nutritional plasticity based on food availability or environmental pressure.

δ^13^C_H–S_ was lower in the D‐F treatment; however, it appears incorrect to interpret this trend as a sign of greater heterotrophy due to the fact that host and symbionts δ^13^C values were highest in this treatment (−16.6 to −15.1‰) and did not approach δ^13^C values of the plankton (−21.8‰). Therefore, higher δ^13^C values in the D‐F treatment corresponded with lower C : N, which together suggest corals (and Symbiodiniaceae in particular) may have experienced a decline in lipid biomass. Perturbed light : dark cycles can affect coral circadian clock genes (Hoadley et al. 2011) and prolonged darkness can cause behavior changes or energetic deficits that influence polyp extension, feeding rates, and tissue biomass (Clayton and Lasker 1982). In the temperate coral, *Astrangia poculata* polyp extensions were higher in fed vs. unfed corals in both symbiotic and aposymbiotc corals (Burmester et al. 2018), and in darkness *Cladocora caespitosa* corals had lower feeding rates and reduced lipid biomass compared to illuminated treatments (Hoogenboom et al. 2010). In tropical corals, darkness can also cause lower prey ingestion rates and reduce biomass (Clayton and Lasker 1982), which may be due to the host's requirement for autotrophic energy to support the capture and ingestion of prey (Leal et al. 2015). In the context of the present study, changes in bulk δ^13^C values likely relate to changes in tissue composition and the substrates used in metabolism, rather than changes in feeding modes (Swart et al. 2005; Wall et al. 2019). The absence of detectable changes in heterotrophy in treatments where *M. capitata* were fed (L‐F, D‐F) emphasizes that laboratory *M. capitata* exhibits a fidelity to autotrophic nutrition despite changes in food availability, a potential for prey capture to be dependent on symbiont photosynthesis, and the importance of autotrophy and photoacclimation as factors shaping the depth distribution (Wall et al. 2020) and physiological responses of *M. capitata* to environmental stress.

The lack of treatment effects across analyses of bulk tissue and amino acids provide consistent evidence for poor capacity for trophic plasticity in *M. capitata*; yet, heterotrophy is important to coral energy budgets (Houlbrèque and Ferrier‐Pagès 2009) and *M. capitata* does consume plankton (Grottoli et al. 2006). Indeed, *M. capitata* percent heterotrophy calculations based on TP_Glx–Phe_ indicate 1–41% of nitrogen from sources supplemental to symbiont translocation (Supporting Information Table S6); however, higher percent heterotrophy did not correlate with prey availability or the absence of light. This apparent paradox may be explained by a combination of limited contributions of heterotrophy (relative to autotrophy) to holobiont amino acid biosynthesis, the translocation (and recycling) of amino acid building blocks between symbiont partners, and the combined capacity of the coral holobiont to obtain amino acids through diet/assimilation and *de novo* synthesis (*see* below). Bulk tissue δ^15^N and δ^13^C values can vary substantially among coral colonies of the same species in response to symbiont communities (Wall et al. 2020) and spatial scales (meters to kilometers) due to environmental heterogeneity (i.e., water flow, light, upwelling) (Teece et al. 2011; Fox et al. 2019; Radice et al. 2019; Wall et al. 2019), which may affect feeding efforts (Palardy et al. 2005) and the ability to detect trophic changes (Fox et al. 2019). In the present study, colonies differed in their distance to the patch reef slope (and deeper lagoon water). We expect the close proximity (ca. 5 m) and similar depth (< 1 m) of corals collected in the present study likely minimized effects of environmental influences on bulk isotope values prior to coral collections. To what degree coral amino acid isotope values differ in response to host and/or symbiont biological traits vs. environmental conditions is uncertain. Therefore, testing of colony‐level variability (linked to host genotypes, symbiont communities) and environmental influences on amino acid stable isotope values should be further examined under field and laboratory conditions.

### Essential amino acid isotope fingerprinting

Our analysis of carbon and nitrogen isotope values in amino acids show significant overlap in *M. capitata* host and symbiont amino acid isotope fingerprints in both PC and linear discriminant plots (Figs. 2, 4). In particular, δ^13^C_AA_ values are similar for both essential and nonessential amino acids, indicating a shared source of carbon for host and symbiont amino acids biosynthesis. Surprisingly, the PCA showed it was the presence or absence of light (and not feeding) that produced the largest differences in δ^13^C fingerprints (Fig. 2c), indicating a significant influence of symbiont photosynthesis on coral essential amino acids. However, when examining δ^13^C values and differences in tissue fractions and treatment effects (Supporting Information Table S4), we show only glutamic acid and glycine δ^13^C (both nonessential‐AA) differed between host and symbiont tissue fractions, with only glycine being influenced by nutrition treatments (Supporting Information Fig. S1c). The most reasonable explanation of these trends appears to be that *M. capitata* uses the same proportion of dietary carbon sources (i.e., autotrophy : heterotrophy) and most, if not all, amino acids may be synthesized and shared within the coral holobiont with minimal direct contributions from allochthonous nutrition. Tissue isotope values integrate nutritional signals over long periods; therefore, the absence of nutrition treatment effects (δ^13^C and δ^15^N) in the present study (1 month) may also be a result of tissue turnover rates in the host and symbiont (Tanaka et al. 2018; Rangel et al. 2019) and different assimilation (and metabolism) of carbon and nitrogen derived from autotrophic vs. heterotrophic nutrition (Krueger et al. 2018). Nitrogen turnover time in particular may be prolonged (2–12 months) and differ between host and symbiont tissues and the inputs from dissolved or heterotrophic nitrogen sources (Tanaka et al. 2018; Rangel et al. 2019). Therefore, more and potentially longer manipulative feeding studies are required to determine how AA‐CSIA differ among colonies with different environmental histories (i.e., depth and light environment) and to test for the influence of tissue turnover times and colony‐level traits (physiology, genetic) on AA‐CSIA data.

While there are limited δ^13^C_AA_ data for essential and non‐essential amino acids in corals and heterotrophic sources (Fox et al. 2019), our results align well with earlier findings. Notably, essential and nonessential‐δ^13^C_AA_ values show significant overlap between hosts and symbionts, with most amino acids in hosts and symbionts being enriched in ^13^C relative to those amino acids in the plankton‐POM. In addition, corals and Symbiodiniaceae showed greater separation from plankton for essential‐δ^13^C_AA_ values, in particular for isoleucine, lycine, and threonine (this study, Fox et al. 2019). Capturing the variability in amino acid isotope values of heterotrophic sources (i.e., POM, range of plankton size classes) was beyond the scope of the present study. However, bulk δ^13^C and δ^15^N values across a range of plankton size classes (including those used here, 63–250 *μ*m) in Kāne'ohe Bay show little spatiotemporal variance (Supporting Information Table S1, *see* also Wall et al. 2020). In Palmyra, where δ^13^C and δ^15^N values in zooplankton communities also show little difference among pelagic and lagoonal habitats, δ^13^C values of essential and nonessential amino acids did not differ between plankton and POM (Fox et al. 2019). Therefore, in the absence of significant spatiotemporal variation in plankton community composition and constitutive biochemical pathways, amino acid isotope values may be stable at the base of the food web. Therefore, essential‐δ^13^C_AA_ appear to be particularly useful in testing for trophic interactions linked to colony‐level changes in feeding behavior and/or environmental factors (Teece et al. 2011; Fox et al. 2018; Radice et al. 2019), but more tests are needed to evaluate sources of variability in amino acid isotope values (and food quality) in coastal marine food webs and coral reef ecosystems (McMahon et al. 2015).

Identifying the sources of essential amino acids in *M. capitata* was enabled by (i) using linear discriminant analysis of mean‐normalized essential‐δ^13^C_AA_ values (isoleucine, leucine, lysine, phenylalanine, threonine, tyrosine, valine); (ii) leveraging previously published δ^13^C_AA_ values of coral‐Symbiodiniaceae and heterotrophic food sources (McMahon et al. 2015; Fox et al. 2019); (iii) and comparing Symbiodiniaceae isolated from corals in Hawai'i and Palmyra to a larger collection of natural zooplankton assemblages and cultured microalgae (Hannides et al. 2013; Larsen et al. 2013). Evaluating these data sets revealed several compelling findings. First, PCA revealed considerable overlap between microalgae and zooplankton mean‐normalized essential‐δ^13^C_AA_ fingerprints (Supporting Information Fig. S5a), and when Symbiodiniaceae were added to the data matrix their δ^13^C_AA_ fingerprint was unique compared to microalgae and zooplankton (Supporting Information Fig. S5b). While there is variability in plankton end‐members that requires further analysis, nevertheless, the consistent difference in Symbiodiniaceae related to plankton suggests such essential‐δ^13^C_AA_ fingerprints are promising for detecting differences between autotrophic and heterotrophic food sources (i.e., Symbiodiniaceae‐derived vs. prey capture) in corals across space and time. Using plankton, coral hosts, Symbiodiniaceae, and a coral holobiont (host+symbiont), we find similar overlap in plankton samples (Hawai'i, Palmyra, Red Sea) (Fig. 4b), which were distinct from those of Symbiodiniaceae (Hawai'i, Palmyra). Coral holobionts from the Red Sea, like those from coral hosts in Hawai'i, overlapped with essential‐δ^13^C_AA_ fingerprints of the symbionts and showed limited overlap with plankton, whereas Palmyra corals were equally spread between symbiont and plankton groups (Supporting Information Fig. S4a,b). Overall, these data show that plankton samples have conserved essential‐δ^13^C_AA_ fingerprints that are different from those in the Symbiodiniaceae symbionts and the essential‐δ^13^C_AA_ fingerprints of corals reflect the proportions of these food sources coral hosts consume, which varies according to coral species, locations, or both.

Using the analytical approach employed by Fox et al. (2019) of essential‐δ^13^C_AA_ fingerprints of six essential amino acids in isolated coral hosts and Symbiodiniaceae symbionts, we found LDA to be effective in assigning corals to food sources based on the proportions of these sources to animal diets. The Hawai'i plankton sample was the approximate centroid for the probability ellipse of the larger group of heterotrophic food sources from Palmyra (Fox et al. 2019) and again provides support for using mean‐normalized essential‐δ^13^C_AA_ to effectively compare food sources across spatial scales (Fig. 4) (Larsen et al. 2020). Coral hosts generally grouped with their respective Symbiodiniaceae symbionts, but holobiont‐specific patterns in essential‐δ^13^C_AA_ values were apparent, suggesting differences in biosynthetic capacities and/or feeding modes. We found all *M. capitata* corals grouped with autotrophic (symbiont) nutrition, whereas *P. meandrina* showed trophic plasticity (41% of essential amino acid carbon from heterotrophy; Fox et al. 2019) and grouped with both symbiont and plankton nutritional sources. Therefore, our results using individual amino acids, essential‐δ^13^C_AA_ fingerprints, and feeding manipulation consistently show *M. capitata* to be largely reliant on autotrophic nutrition for essential amino acid biosynthesis. Considering the limitations in available data for AA‐CSIA, it is important to continue verifying isotope fingerprint approaches among coral species and reef locations. In this task, linear discrimant analyses of mean‐normalization of essential‐δ^13^C_AA_ fingerprints appear promising for understanding coral trophic ecology and testing nutritional plasticity.

### Amino acid nitrogen isotope values and trophic position

Trophic amino acids undergo transamination and reflect trophic connections between dietary sources and consumers, whereas source amino acids show minimal isotopic discrimination between consumers and their diet and propagate through food webs with minimal bond‐breakage. Measured differences between phenylalanine in both *M. capitata* host and Symbiodiniaceae fractions relative to plankton were small and glutamic acid values overlapped for the host and symbiont, indicating a shared position as the base of the food web (Supporting Information Fig. S1). Our values are similar to those reported for the coral *Acropora digitifera* from Japan and identify corals and their symbionts as having a trophic position of ~ 1—a value indicative of primary producers. In a larger sampling of corals from Japan, TP_Glx–Phe_ in host and symbionts ranged from 0.8 to 1.4 and was indicative of a range of heterotrophic dependence for nitrogen (<27% heterotrophy). Host TP_Glx–Phe_ was also positively correlated with both bulk symbiont δ^15^N values and TP_Glx–Phe_, which was interpreted as a sign of enhanced feeding in polluted nearshore habitats (Fujii et al. 2020). However, corals may not shift trophic positions even across extreme environmental change. For instance, *Stylophora pistillata* mean TP_Glx–Phe_ was similar in mesophotic and shallow hosts and symbionts (1.3–1.7); thus, heterotrophy represented the same proportion of energy to *S. pistillata* metabolism (~ 35%) across a range of habitats and environmental conditions (Martinez et al. 2020).

We show *M. capitata* are autotrophic (TP_Glx–Phe_ = 1) and the most abundant heterotrophic food source was likely microalgae‐derived detritus and/or digested symbiont cells (TP_Glx–Phe_ = 1), with contributions from zooplanktivory being small (TP_Glx–Phe_ = 2). Interestingly, *M. capitata* mean percent heterotrophy was lower in fed‐treatments (1–6%) compared to the Light‐Not Fed treatment (heterotrophy 21–41%). Considering this effect was observed in both genotypes used in the study, treatment effects on TP_Glx–Phe_ and percent heterotrophy may be greater than colony‐specific attributes related to feeding effort. While intriguing, due to the low statistical power (small effect size and low replication) and no difference in either TP_Glx–Phe_ or the difference in the δ^15^N‐weighted mean values for trophic and source amino acids of hosts and symbionts, caution is required in interpreting these percent heterotrophy calculations from TP_Glx–Phe_. However, these results suggest corals feed less often on prey of TP_Glx–Phe_ of 2.0 and microalgae‐derived detritus, and not zooplanktivory, is the dominant source of heterotrophy identified in previous studies using these calculations (Fujii et al. 2020; Martinez et al. 2020). As more AA‐CSIA data sets become available for coral and invertebrates (Shih et al. 2020), it will be important for fundamental assumptions in these methods to be evaluated (i.e., canonical constants, enrichment factors, prey sources) as these may vary in response to diet quality or due to underlying differences in the potential for corals and their Symbiodiniaceae or microbiomes to synthesize amino acids and demonstrate nutritional flexibility or fidelity.

Using the trophic‐δ^15^N_AA_ fingerprint in PC plots, we observed trophic‐δ^15^N_AA_ values in the host tended to be higher than those in the symbiont, and source‐δ^15^N_AA_ values in the plankton were substantially higher (up to 7‰) than both host and symbiont. However, the single sample of the plankton end‐member in the present study limits the inferences of patterns in corals relative to the plankton. Nevertheless, similarities in trophic‐ and source‐δ^15^N_AA_ values for both host and symbiont tissue fractions indicate a shared source of amino acid nitrogen and the influence of holobiont nitrogen conservation and/or recycling (Falkowski et al. 1984). Dissolved nitrogen is rapidly assimilated by Symbiodiniaceae (< 1 h) and transferred to the host (~ 6 h), presumably in the form of amino acids and glycoconjugates (Kopp et al. 2013). Similarly, dietary end products (i.e., ${\mathrm{NH}}_{4}^{+}$ and CO_2_) of host planktivory are assimilated and recycled by Symbiodiniaceae, with <23% of heterotrophic carbon and nitrogen pools being assimilated by the symbionts in <6 h (Krueger et al. 2018). Over longer periods, host metabolism can supply up to 80% of Symbiodiniaceae nitrogen (Tanaka et al. 2015). In the context of our study, the large difference in δ^15^N_AA_ values between *M. capitata* (host and symbionts) and the plankton, particularly for those amino acids predicted to show connections between diet and consumers (i.e, trophic‐δ^15^N_AA_), does not support a significant role of zooplanktivory in the diet of *M. capitata* or as a significant source of nitrogen in amino acid biosynthesis. Instead, we suggest a dominant influence of *de novo* biosynthesis of trophic and source amino acids by the host and/or Symbiodiniaceae in the *M. capitata* holobiont, with the possibility for a significant yet variable influence of low trophic position food sources (e.g., digested symbiont cells, microalgae) on trophic amino acids.

The role of microbial symbionts in coral amino acids is uncertain. To test for microbial influences on δ^15^N_AA_ values, we applied an isotope proxy indicative of reworking of organic matter by heterotrophic bacteria and amino acid resynthesis based on trophic‐δ^15^N_AA_ values (i.e., ∑V) (McCarthy et al. 2007). ∑*V* values for *M. capitata* host and symbiont tissues were below those reported for the sponge *Mycale grandis* near southern Kāne'ohe Bay (isolated sponge‐cells and associated microbes ∑*V* range 2.3–3.0). In this case, higher ∑*V* in *M. grandis*, indicated microbial symbionts as a source for translocated amino acids (Shih et al. 2020). We found ∑*V* values were low (~ 1) and resembled those reported for oceanic zooplankton (McCarthy et al. 2007) and did not differ significantly in host and symbiont tissues or among treatments (Supporting Information Fig. S2b). ∑*V* calculations may differ depending on the number of trophic amino acids included in calculations; nevertheless, we show microbial resynthesis of amino acids does not appear to drive patterns in coral trophic‐δ^15^N_AA_ values.

### Sources of amino acids: A combination of host and symbiont biosynthesis

CSIA may provide new insights into the trophic ecology of reef corals and clarify trends observed in bulk tissue isotope analyses; however, questions regarding the source of amino acids in reef corals remain unanswered. Symbiotic anthozoans are able to acquire many amino acids from their endosymbiont Symbiodiniaceae (Markell and Trench 1993). For instance, symbiont fixed ^14^C was tracked into anemone and zoanthid host protein and constituent amino acids (alanine, glutamic acid) (Trench 1971*a*, *b*) and was also observed in seven amino acids in the proteins of *Aiptasia pulchella* (Wang and Douglas 1999). The release of glycoconjugates rich in essential amino acids by freshly isolated Symbiodiniaceae also suggests Symbiodiniaceae as a source for cnidarian host amino acids (Markell and Trench 1993). The genome of *Symbiodinium kawagutti* (formerly of Clade F) (LaJeunesse et al. 2018) further reveals that this endosymbiont contains complete biosynthesis pathways for nine amino acids (except lysine and histidine) and may supply these to its coral host (*Acropora digitifera*), which lacks these pathways (Lin et al. 2015).

The role of the host is not insignificant, however, and cnidarians are capable of *de novo* synthesis of essential amino acids. Incubating five symbiotic and nonsymbiotic scleractinian corals with ^14^C‐radiolabeled amino acid precursors (glucose, glutamic acid) and tricarboxylic acid cycle intermediates (lysine, valine) showed scleractinians can synthesize 16 of 20 protein amino acids, eight of which are considered essential (Fitzgerald and Szmant 1997). In a separate study, *Aiptasia pulchella* not only acquired the majority of amino acids from its endosymbionts, but also synthesized methionine and threonine (Wang and Douglas 1999). Recent genomic evidence, however, is revealing diversity in the capacity of cnidarians to synthesize amino acids. For instance, reef corals in the *Robusta* clade have a complete histidine biosynthesis pathway, but this pathway may be absent within the *Complexa* clade and sea anemones (Ying et al. 2018). Therefore, genomic studies appear to be a promising avenue to distinguish origins of amino acids and the diverse capacities for amino acid biosynthesis within the Cnidaria and Symbiodiniaceae.

## Conclusion

Our findings using AA‐CSIA cannot distinguish between the *de novo* synthesis of amino acids by the host or the translocation of amino acids from the symbiont to the host. However, our data provide new insights into the amino acid biosynthesis pathways and their relationship to coral trophic ecology. First, coral and Symbiodiniaceae δ^13^C values in essential and nonessential amino acids were highly similar to each other, yet distinct from those in the plankton. These findings indicate heterotrophy offers a limited contribution to *M. capitata* holobiont amino acids and modifying this contribution (i.e., through experimental nutrition treatments) may be difficult to inexorable—a finding further supported by lack of clear nutrition treatment effects on TP_Glx–Phe_ calculations. The apparent trophic fidelity in *M. capitata* under experimental nutrition regimes contrasts with the flexibility observed in *P. meandrina* colonies *in situ* (Fox et al. 2019). Therefore, trophic plasticity may be determined by species‐specific traits (behavior, morphologies) or genomic capabilities for amino acid biosynthesis. Second, in a similar pattern as observed with carbon, δ^15^N_AA_ values rarely differed between host and symbiont fractions, but δ^15^N_AA_ fingerprints suggest an influence of planktivory on trophic‐δ^15^N_AA_ values to a greater extent than source‐δ^15^N_AA_ values, although this effect is small. δ^15^N values of phenylalanine in all members (host, symbiont, plankton) are quite similar. Thus, TP_Glx–Phe_ calculations may be problematic in the absence of large shifts in heterotrophically derived glutamic acid, which we did not observe despite considerable differences in treatment conditions. Finally, low TP_Glx–Phe_ and ∑*V* values suggest corals exist at a lower trophic position than zooplankton (indicating greater feeding on microalgae detritus compared to zooplanktivory), and there is no clear influence of microbial processing of dissolved organic matter and microbial amino acid resynthesis in corals or Symbiodiniaceae δ^15^N_AA_ values. Corals feed on a variety of plankton size classes, and heterotrophic nutrition can vary due to biological and behavioral attributes (i.e., polyp size, physiological state, feeding effort) and across environments (Palardy et al. 2005). Therefore, as the application of AA‐CSIA expands in coming years, it will be important for ecological studies to be matched with manipulative feeding studies of varying diet type and quality using corals with different emergent properties.

## Conflict of Interest

None declared.

## Supporting information


**Appendix S1:** Supporting information.

## Data Availability

All data and code to generate figures and perform analyses are archived and openly available at GitHub (https://github.com/cbwall/CSIA-corals) and archived at Zenodo (http://doi.org/10.5281/zenodo.4527785).
