# Supplementary material for: Amino acid δ13C and δ15N analyses reveal distinct species‐specific patterns of trophic plasticity in a marine symbiosis
Source: Limnol Oceanogr. 2021 Apr 7;66(5):2033–50. doi: 10.1002/lno.11742 (PMC8252108; doi:10.1002/lno.11742)
Supplement: Supplementary file 1 — Appendix S1: Supporting information. [file LNO-66-2033-s001.pdf]

Amino acid $\delta^{13}C$ and $\delta^{15}N$ analyses reveal distinct species-specific patterns of trophic plasticity in a marine symbiosis

Christopher B. Wall*, Natalie J. Wallsgrove, Ruth D. Gates, Brian N. Popp

*Corresponding author: chris.wall@hawaii.edu

Supplemental Information

Bulk tissue isotope analysis

Sample analytical accuracy and precision ($\delta^{13}C$ and $\delta^{15}N$) was <0.2 ‰ based on repeated analysis of laboratory reference material (glycine and a homogenate of tuna white muscle tissue) run before and after every 10 samples. Quantification and isotopic corrections were calculated using the glycine laboratory reference material run alongside samples, and the isotopic composition of the glycine was characterized using international reference materials including USGS 32, USGS 34, USGS 35, IAEA N3, USGS 40, USGS 41, NBS 18 and NBS 19. The known values for glycine are +11.36 ‰ vs. AIR for $\delta^{15}N$, and -36.49 ‰ vs. V-PDB for $\delta^{13}C$.

Individual amino acid isotope analyses

Isotopic analysis of amino acids in coral host, symbiont algae, and a pooled plankton sample were performed by subjecting tissue samples to acid hydrolysis, carboxyl terminus esterification, and amine group trifluoroacetylation (Hannides et al. 2013; Shih et al. 2020). Acid hydrolysis was performed by heating (150 °C) tissues (approx. 15 mg) in 6N HCl for 70 min in a culture tube fitted with a Teflon-lined cap and filled with N_2 gas. HCl was evaporated to dryness, hydrolysate redissolved in 0.01N HCl, and filtered through a 0.2 µm polyethersulfone filter with a 0.01N HCl wash. The hydrolysate solubilized in 0.01N HCl was purified by cation exchange

(Dowex 50WX8-400), and amino acids were eluted with ammonium hydroxide. Hydrolyzed tissues were esterified by heating (110 °C) for 60 min in 1:4 acetyl chloride : isopropanol and the amine group was trifluoroacetylated by adding 3:1 methylene : trifluoroacetic anhydride (TFAA) solution to vials and heating (100 °C) for 15 min. Finally, solvent extraction in P-buffer ($KH_2PO_4 + Na_2HPO_4$ in milli-Q water, pH 7) was used to further purify samples (Ueda et al. 1989). Chloroform was used to partition acylated amino acids, and following solvent evaporation, sample trifluoroacetylation was repeated to maximize derivitization. Samples were stored frozen in 3:1 methylene chloride : TFAA at -20 °C until analyzed.

Trophic position calculations

While values for β and Δ have been widely used in the study of a marine invertebrates and fishes to calculate trophic positions (Chikaraishi et al. 2009; Hannides et al. 2013; Shih et al. 2020) and more recently in reef corals (Fujii et al. 2020), we tested the applicability of the canonical β and Δ values for $TP_{Glx\text{-}Phe}$. The β values calculated for isolated Symbiodiniaceae (3.7 ± 0.8 ‰, propagated error, $n = 6$) were similar to that found by (Fujii et al. 2020) and not statistically different from 3.4 ± 1.0 ‰ (p=0.407). In addition, the average trophic position for Symbiodiniaceae calculated using equation 1 is not different from 1 (1.04 ± 0.22), which correctly identifies Symbiodiniaceae as a primary producer and indicates that the $\Delta_{Glx\text{-}Phe}$ value of 7.6 ‰ is applicable for this alga. In other works, trophic positions in Symbiodiniaceae and hermatypic corals have been reported to range from 0.7-1.5 and 0.8-1.8, respectively (Fuji et al. 2020; Martinez et al. 2020), with trophic positions in temperate heterotrophic cnidarians (sea jellies, octocorals) ranging from 2.2-3.7 (Grossowicz et al. 2020).

Supplemental Tables

Table S1. Bulk isotope samples in plankton and suspended particles in seawater adjacent to six reefs spanning Kāne‘ohe Bay in August and December 2016. Isotopes values in the pooled plankton sample used in the present study are presented for comparison (collected January 2018).

Size fraction (µm)	$\delta^{13}C$	$\delta^{15}N$
<10	-21.04 ± 0.79	5.33 ± 0.14
10-100	-18.06 ± 0.44	7.41 ± 0.12
100-243	-21.06 ± 0.19	6.50 ± 0.11
<243	-20.44 ± 0.50	5.92 ± 0.17
>243[a]	-20.75 ± 0.28	6.77 ± 0.22
63-243[b]	-21.8	7.1

Values are mean±SE (*n*=12), except [b] = pooled plankton sample (*n*=1, this study). All samples were filtered from seawater collected at 3 m depth, except [a,b] = collected from vertical and horizontal plankton tows (63 µm mesh) (data from Wall et al., 2020).

Table S2. Statistical analyses of carbon and nitrogen isotope values ($\delta^{13}C$, $\delta^{15}N$) in bulk tissues of coral and Symbiodiniaceae exposed to Light-by-Feeding nutrition treatments*.

Bulk tissue isotope values	*Effect*	*df*	*SS*	*MS*	*F*	*p*
$\delta^{13}C$	Fraction	1	1.401	1.401	1.532	0.251
	Treatment	2	3.772	1.886	2.062	0.190
	Residual	8	7.317	0.915		
$\delta^{15}N$	Fraction	1	4.083	4.083	14.770	**0.005**
	Treatment	2	0.105	0.053	0.190	0.831
	Residual	8	2.212	0.277		
C:N	Fraction	1	0.132	0.132	0.723	0.420
	Treatment	2	2.218	1.109	6.086	**0.025**
	Residual	8	1.457	0.182		
$\delta^{13}C_{H-S}$	Treatment	1	0.763	0.382	7.897	0.064
	Residual	2	0.145	0.048		
$\delta^{15}N_{H-S}$	Treatment	1	0.063	0.032	0.288	0.769
	Residual	2	0.330	0.110	0.000	

*'Fraction' is host coral (H) or symbiont Symbiodiniaceae tissue (S). 'Treatment' represents combination of Light-by-Feeding nutrition treatments: Light–Not Fed, Light–Fed, Dark–Fed. Significant p-values (p<0.05) are in bold.

Table S3. Carbon and nitrogen isotope values ($\delta^{13}C$, $\delta^{15}N$) of amino acids measured in pooled samples of coral host (*Montipora capitata*) and endosymbiont algae (Symbiodiniaceae) along with a size-fractioned plankton sample*.

Amino acid $\delta^{13}C$ (‰)	*Coral*	*Symbiodiniaceae*	*Pooled plankton*
Alanine (Ala)	-15.35 ± 0.29	-16.98 ± 0.66	-20.83
Aspartic acid (Asp)	-10.35 ± 0.39	-9.55 ± 0.69	-15.38
Glutamic acid (Glx)	-8.11 ± 0.54	-10.38 ± 0.47	-16.12
Glycine (Gly)	-13.44 ± 0.66	-17.25 ± 1.26	-19.48
Isoleucine (Ile)	-14.21 ± 0.59	-15.09 ± 0.93	-21.81
Leucine (Leu)	-24.69 ± 0.43	-24.94 ± 0.45	-27.84
Lysine (Lys)	-11.59 ± 0.33	-12.08 ± 0.56	-19.34
Phenylalanine (Phe)	-19.94 ± 0.50	-20.34 ± 0.42	-24.88
Proline (Pro)	-10.32 ± 0.26	-12.16 ± 0.73	-17.28
Serine (Ser)	-9.13 ± 0.70	-8.72 ± 0.81	-11.03
Threonine (Thr)	-10.53 ± 1.01	-9.67 ± 0.70	-17.90
Tyrosine (Tyr)	-21.08 ± 0.61	-20.70 ± 0.53	-25.22
Valine (Val)	-24.26 ± 0.55	-25.27 ± 0.69	-27.20
Amino acid $\delta^{15}N$ (‰)			
Alanine (Ala)	7.02 ± 0.47	5.37 ± 0.69	12.53
Aspartic acid (Asp)	6.44 ± 0.36	5.38 ± 0.25	8.94
Glutamic acid (Glx)	6.38 ± 0.23	5.77 ± 0.33	11.55
Glycine (Gly)	2.84 ± 0.58	4.03 ± 0.63	5.60
Isoleucine (Ile)	6.26 ± 0.79	5.93 ± 0.70	7.25
Leucine (Leu)	4.65 ± 0.41	2.82 ± 0.30	6.72
Lysine (Lys)	3.08 ± 0.28	2.89 ± 0.53	3.84
Phenylalanine (Phe)	1.35 ± 0.38	2.03 ± 0.76	0.57
Proline (Pro)	6.81 ± 0.43	4.05 ± 0.41	11.99
Serine (Ser)	3.73 ± 0.58	3.72 ± 0.48	6.05
Threonine (Thr)	-1.06 ± 0.77	1.04 ± 0.93	-1.98
Tyrosine (Tyr)	2.11 ± 0.35	4.43± 0.60	4.62
Valine (Val)	6.24 ± 0.42	5.97 ± 0.32	9.79

*Coral and Symbiodiniaceae values are pooled across three nutrition treatments, which had limited effects on carbon and nitrogen isotope values. Values are mean ± SE (n = 6), except for the pooled plankton sample (63 – 250 µm) (n = 1).

Table S4. Statistical analyses of carbon isotope values ($\delta^{13}C$) in coral and Symbiodiniaceae amino acids exposed to Light-by-Feeding nutrition treatments*.

Amino acid $\delta^{13}C$	*Effect*	*df*	*SS*	*MS*	*F*	*p*
Nonessential amino acids						
Alanine (Ala)	Fraction	1	7.928	7.928	4.849	0.059
	Treatment	2	2.386	1.193	0.730	0.512
	Residual	8	13.080	1.635		
Aspartic acid (Asp)	Fraction	1	1.937	1.937	0.870	0.378
	Treatment	2	1.110	0.555	0.249	0.785
	Residual	8	17.798	2.225		
Glycine (Gly)	Fraction	1	43.514	43.514	16.281	**0.004**
	Treatment	2	38.910	19.455	7.279	**0.016**
	Residual	8	21.382	2.673		
Glutamic acid (Glx)	Fraction	1	15.418	15.418	15.500	**0.004**
	Treatment	2	7.177	3.589	3.608	0.076
	Residual	8	7.958	0.995		
Proline (Pro)	Fraction	1	10.096	10.096	5.023	0.055
	Treatment	2	1.963	0.981	0.488	0.631
	Residual	8	16.081	2.010		
Serine (Ser)	Fraction	1	0.510	0.510	0.191	0.674
	Treatment	2	13.333	6.667	2.498	0.144
	Residual	8	21.353	2.669		
Tyrosine (Tyr)	Fraction	1	0.427	0.427	0.220	0.652
	Treatment	2	3.794	1.897	0.974	0.418
	Residual	8	15.577	1.947		
Essential amino acids						
Isoleucine (Ile)	Fraction	1	2.301	2.301	0.509	0.496
	Treatment	2	0.536	0.268	0.059	0.943
	Residual	8	36.157	4.520		
Leucine (Leu)	Fraction	1	0.181	0.181	0.146	0.713
	Treatment	2	1.819	0.910	0.734	0.510
	Residual	8	9.913	1.239		
Lysine (Lys)	Fraction	1	0.715	0.715	0.458	0.518
	Treatment	2	0.083	0.041	0.027	0.974
	Residual	8	12.498	1.562		
Phenylalanine (Phe)	Fraction	1	0.472	0.472	0.340	0.576
	Treatment	2	1.717	0.858	0.619	0.562
	Residual	8	11.093	1.387		
Threonine (Thr)	Fraction	1	2.197	2.197	0.446	0.523
	Treatment	2	5.526	2.763	0.561	0.591
	Residual	8	39.372	4.922		
Valine (Val)	Fraction	1	3.055	3.055	1.273	0.292
	Treatment	2	4.477	2.239	0.933	0.432
	Residual	8	19.199	2.400		

*'Fraction' is host coral or symbiont Symbiodiniaceae tissue. 'Treatment' represents combination of Light-by-Feeding nutrition treatments: Light–Not Fed, Light–Fed, Dark–Fed. Significant p-values ($p<0.05$) are in bold.

Table S5. Statistical analyses of nitrogen isotope values ($\delta^{15}N$) in coral and Symbiodiniaceae amino acids exposed to Light-by-Feeding nutrition treatments*.

Amino acid $\delta^{15}N$	*Effect*	*df*	*SS*	*MS*	*F*	*p*
Trophic amino acids						
Alanine (Ala)	Fraction	1	8.168	8.168	3.392	0.103
	Treatment	2	1.858	0.929	0.386	0.692
	Residual	8	19.262	2.408		
Aspartic acid (Asp)	Fraction	1	3.333	3.333	6.317	**0.036**
	Treatment	2	1.420	0.710	1.346	0.314
	Residual	8	4.221	0.528		
Glutamic acid (Glx)	Fraction	1	1.115	1.115	2.361	0.163
	Treatment	2	0.935	0.467	0.990	0.413
	Residual	8	3.777	0.472		
Isoleucine (Ile)	Fraction	1	0.338	0.338	0.091	0.771
	Treatment	2	3.858	1.929	0.518	0.615
	Residual	8	29.800	3.725		
Leucine (Leu)	Fraction	1	9.995	9.995	19.904	**0.002**
	Treatment	2	3.809	1.904	3.792	0.069
	Residual	8	4.017	0.502		
Proline (Pro)	Fraction	1	22.935	22.935	28.936	**0.001**
	Treatment	2	4.117	2.059	2.597	0.135
	Residual	8	6.341	0.793		
Valine (Val)	Fraction	1	0.225	0.225	0.220	0.652
	Treatment	2	0.042	0.021	0.020	0.980
	Residual	8	8.170	1.021		
Source amino acids						
Glycine (Gly)	Fraction	1	4.226	4.226	1.650	0.235
	Treatment	2	1.569	0.785	0.306	0.744
	Residual	8	20.487	2.561		
Lysine (Lys)	Fraction	1	0.105	0.105	0.080	0.785
	Treatment	2	0.245	0.123	0.093	0.912
	Residual	8	10.530	1.316		
Serine (Ser)	Fraction	1	0.001	0.001	0.000	0.986
	Treatment	2	1.661	0.831	0.440	0.659
	Residual	8	15.113	1.889		
Phenylalanine (Phe)	Fraction	1	1.400	1.400	0.536	0.485
	Treatment	2	0.801	0.400	0.153	0.861
	Residual	8	20.916	2.615		
Threonine (Thr)	Fraction	1	13.327	13.327	2.658	0.142
	Treatment	2	3.612	1.806	0.360	0.708
	Residual	8	40.117	5.015		
Tyrosine (Tyr)	Fraction	1	16.163	16.163	8.950	**0.017**
	Treatment	2	0.128	0.064	0.035	0.965
	Residual	8	14.448	1.806		

*'Fraction' is host coral or symbiont Symbiodiniaceae tissue. 'Treatment' represents combination of Light-by-Feeding nutrition treatments: Light–Not Fed, Light–Fed, Dark–Fed. Significant p-values (p<0.05) are in bold.

Table S6. Fractional contribution of heterotrophy (%) to coral nutrition calculated using a simple two component linear mixing model and $\delta^{15}N$ amino acid trophic position ($TP_{Glx\text{-}Phe}$). End-member $TP_{Glx\text{-}Phe}$ used to calculate % heterotrophy are (*i*) *Montipora capitata* coral hosts (*ii*) symbionts isolated from *M. capitata,* (*iii*) a pooled Kāne'ohe Bay plankton sample, and (*iv*) modeled values for marine consumers feeding on zooplankton prey. All uncertainties represent standard deviation of propagated errors.

Treatment	***$TP_{Glx\text{-}Phe}$***				***% Primary consumers [b] vs. Translocation [a]***	***% Zooplanktivory [c] vs. Translocation***
	Coral host	*Symbiont algae*	*Primary consumers*	*Zooplankton consumers*		
Light-Not Fed	1.35 ± 0.17	0.90 ± 0.17	2.00 ± 0.22	3.00 ± 0.20	41 ± 15%	21 ± 7%
Light-Fed	1.13 ± 0.17	1.12 ± 0.16	2.00 ± 0.22	3.00 ± 0.20	1 ± 19%	1 ± 9%
Dark-Fed	1.16 ± 0.16	1.11 ± 0.18	2.00 ± 0.22	3.00 ± 0.20	6 ± 19%	3 ± 9%

[a] *Translocation* $TP_{Glx\text{-}Phe}$ of symbionts = autotrophy, a diet consisting symbiont products
[b] *Primary production* $TP_{Glx\text{-}Phe}$ based on digested symbiont cells, free-living primary producers, and/or allochthonous particulates preyed upon by zooplankton
[c] *Zooplanktivory* $TP_{Glx\text{-}Phe}$ of zooplankton consumers, with trophic enrichment (+1 $TP_{Glx\text{-}Phe}$) of predators *vs.* prey

Supplemental Figures

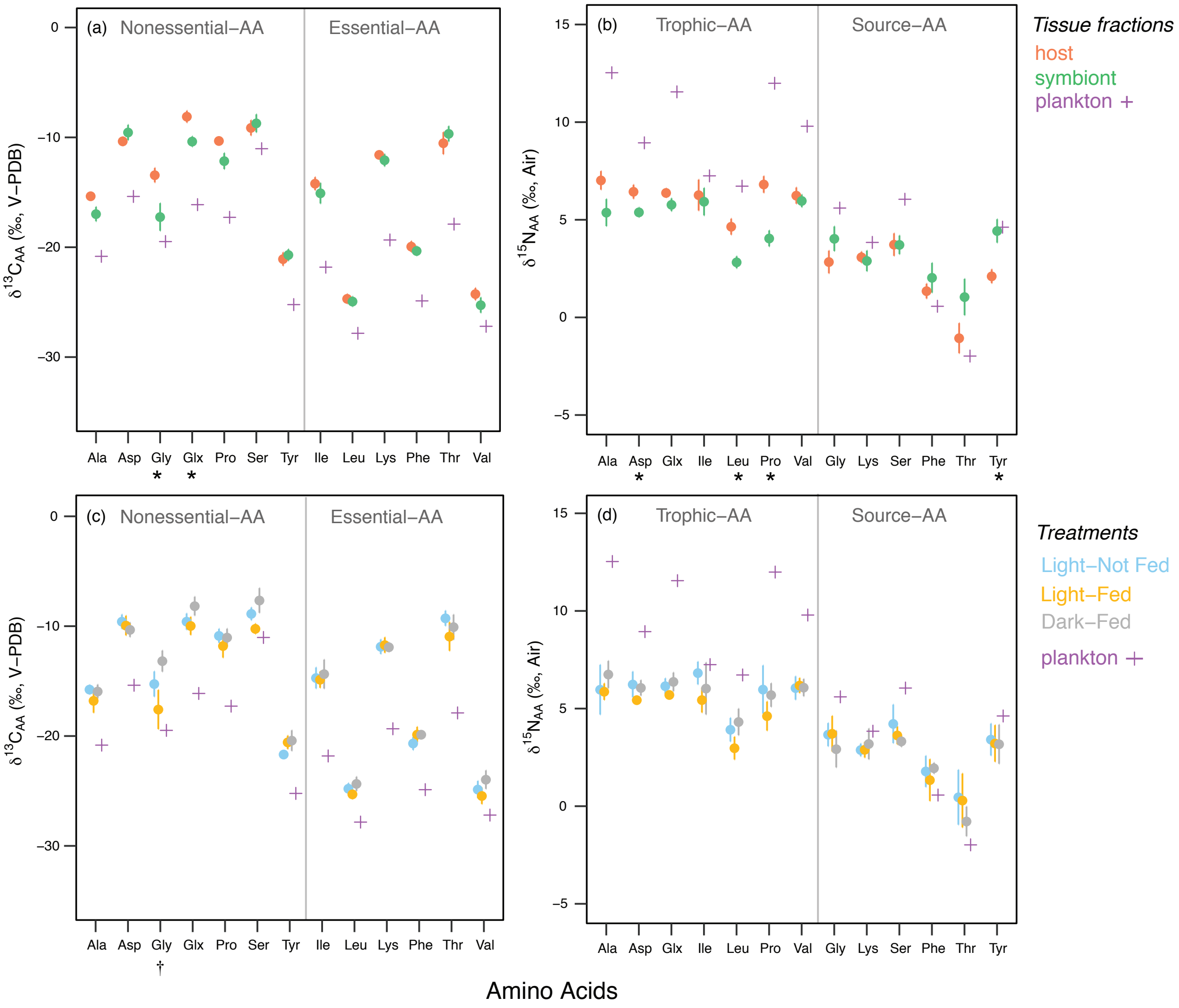

Figure S1. (*a*,*c*) δ^{13}C and (*b*,*d*) δ^{15}N values of individual amino acids in coral hosts and Symbiodiniaceae symbionts according to tissue fraction (*top panel*) and nutrition treatments (*bottom panel*) in relationship to a pooled plankton sample. Values are mean ± SD (*n* = 2) [*top*] and mean ± SE (*n* = 6) [*bottom*], except for the plankton sample (*n* = 1). X-axis *symbols* indicate significant differences (p<0.05) between fractions (host and symbiont, *) and treatments (†).

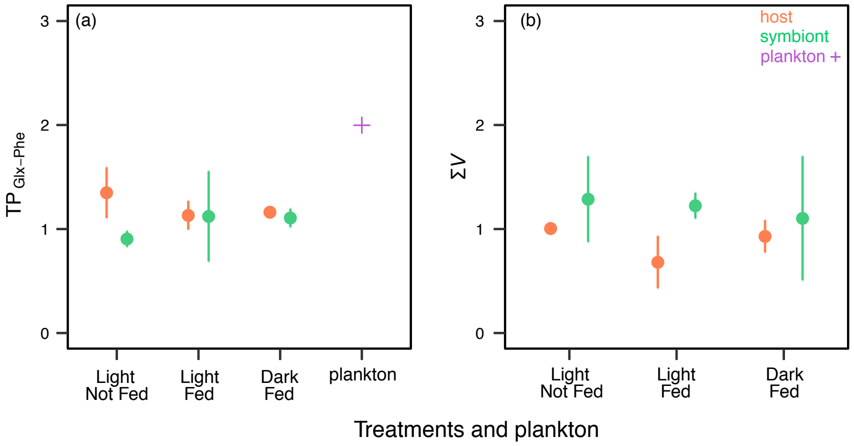

Figure S2. (*a*) Trophic position and (*b*) summed variance (ΣV) index calculations for individual amino acid δ^{15}N values in coral hosts and Symbiodiniaceae symbionts across nutrition treatments in relation to a pooled plankton sample. Values are mean ± SD (n = 2), except for the plankton sample (n = 1), where ΣV cannot be calculated.

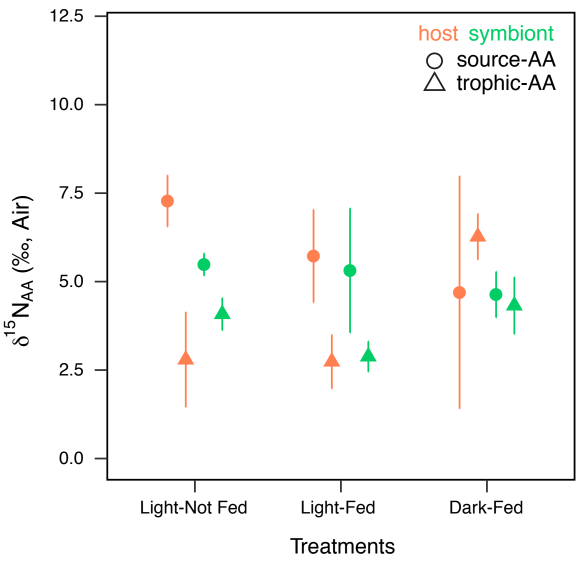

Figure S3. $\delta^{15}N$ weighted means for trophic and source amino acids according to coral tissue fractions (host, symbiont) and experimental nutrition treatments. Values are mean ± SD (n = 2).

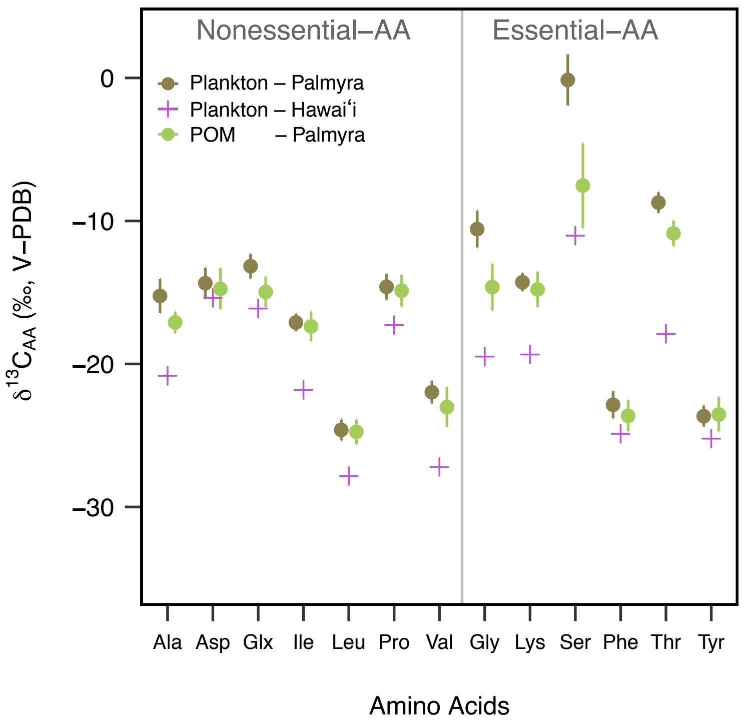

Figure S4. Comparison of $\delta^{13}C$ values for nonessential and essential amino acids in a plankton sample from Kāneʻohe Bay, Oʻahu, Hawaiʻi and plankton and particulate organic material (POM) from Palmyra (reported in Fox et al., 2019). Values are mean ± SE (*n* = 6 – 9), except for the Hawaiʻi plankton (*n* = 1).

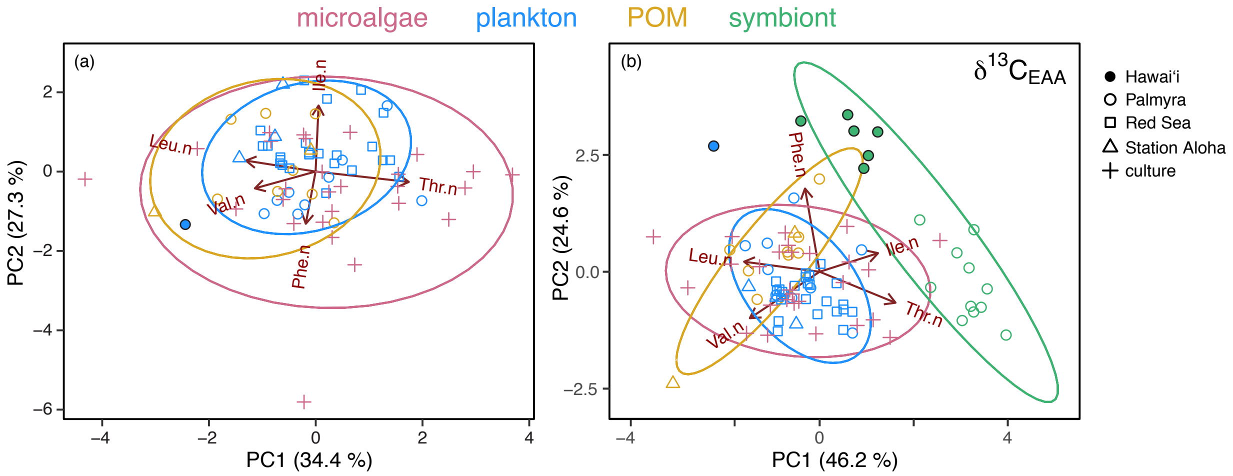

Figure S5. Principal component (PC) analyses of mean-normalized essential amino acid $\delta^{13}C$ ($\delta^{13}C_{EAA}$) values in cultured microalgae, coastal and oceanic plankton and particulate organic matter (POM), and Symbiodiniaceae symbionts isolated from two coral species. Data matrixes in (*a*) and (*b*) are identical, except for the inclusion of symbiont samples in (*b*). Ellipses represent 90 % standard deviation with arrows for individual mean-normalized amino acids being significant ($p<0.05$) correlation vectors. Data from Hawai'i (this study), Palmyra (Fox et al. 2019), the Red Sea (McMahon et al. 2015), Station Aloha (Hannides et al. 2008), and cultures (Larsen et al. 2013).

References

Chikaraishi, Y., N. O. Ogawa, Y. Kashiyama, and others. 2009. Determination of aquatic food-web structure based on compound-specific nitrogen isotopic composition of amino acids: Trophic level estimation by amino acid δ^{15}N. Limnol. Oceanogr. Methods **7**: 740–750. doi:10.4319/lom.2009.7.740

Fox MD, Elliott Smith EA, Smith JE, Newsome SD. 2019 Trophic plasticity in a common reef-building coral: Insights from δ^{13}C analysis of essential amino acids. Funct. Ecol. **33**, 2203–2214. doi:10.1111/1365-2435.13441

Fujii, T., Y. Tanaka, K. Maki, N. Saotome, N. Morimoto, A. Watanabe, and T. Miyajima. 2020. Organic carbon and nitrogen isoscapes of reef corals and algal symbionts: Relative influences of environmental gradients and heterotrophy. Microorganisms **8**: 1221. doi:10.3390/microorganisms8081221

Grossowicz, M., E. Shemesh, S. Martinez, Y. Benayahu, and D. Tchernov. 2020. New evidence of *Melithaea erythraea* colonization in the Mediterranean. Estuar. Coast. Shelf Sci. **236**: 106652. doi:10.1016/j.ecss.2020.106652

Hannides, C. C. S., B. N. Popp, H. G. Close, and others. 2020. Seasonal dynamics of midwater zooplankton and relation to particle cycling in the North Pacific Subtropical Gyre. Prog. Oceanogr. **182**: 102266. doi:10.1016/j.pocean.2020.102266

Larsen, T., M. Ventura, N. Andersen, D. M. O'Brien, U. Piatkowski, and M. D. McCarthy. 2013. Tracing carbon sources through aquatic and terrestrial food webs using amino acid stable isotope fingerprinting. PLoS One **8**: e73441. doi:10.1371/journal.pone.0073441

Martinez, S., Y. Kolodny, E. Shemesh, and others. 2020. Energy sources of the depth-generalist mixotrophic coral *Stylophora pistillata*. Frontiers in Marine Science **7**: 988. doi:10.3389/fmars.2020.566663

McMahon, K. W., S. R. Thorrold, L. A. Houghton, and M. L. Berumen. 2015. Tracing carbon

flow through coral reef food webs using a compound-specific stable isotope approach. Oecologia **180**: 809–821. doi:10.1007/s00442-015-3475-3

Shih, J. L., K. E. Selph, C. B. Wall, N. J. Wallsgrove, M. P. Lesser, and B. N. Popp. 2020. Trophic ecology of the tropical Pacific sponge *Mycale grandis* inferred from amino acid compound-specific isotopic analyses. Microb. Ecol. **79**: 495–510. doi:10.1007/s00248-019-01410-x

Wall, C. B., M. Kaluhiokalani, B. N. Popp, M. J. Donahue, and R. D. Gates. 2020. Divergent symbiont communities determine the physiology and nutrition of a reef coral across a light-availability gradient. ISME J. **14**: 945–958. doi:10.1038/s41396-019-0570-1
